# Effectiveness of attentional bias modification training as add-on to regular treatment in alcohol and cannabis use disorder: A multicenter randomized control trial

**DOI:** 10.1371/journal.pone.0252494

**Published:** 2021-06-04

**Authors:** Janika Heitmann, Madelon E. van Hemel-Ruiter, Mark Huisman, Brian D. Ostafin, Reinout W. Wiers, Colin MacLeod, Laura DeFuentes-Merillas, Martine Fledderus, Wiebren Markus, Peter J. de Jong

**Affiliations:** 1 Verslavingszorg Noord Nederland, Groningen, The Netherlands; 2 Department of Clinical Psychology and Experimental Psychopathology, University of Groningen, Groningen, The Netherlands; 3 Bureau Gedragsstrategie, Assen, The Netherlands; 4 Department of Sociology/ICS, University of Groningen, Groningen, The Netherlands; 5 Addiction Development and Psychopathology (ADAPT)-LAB, Department of Psychology, and Centre for Urban Mental Health, University of Amsterdam, Amsterdam, The Netherlands; 6 School of Psychological Science, The University of Western Australia, Crawley, Australia; 7 Novadic-Kentron, Network for Addiction Treatment Services, Vught, The Netherlands; 8 Stichting Philadelphia Zorg, Amersfoort, The Netherlands; 9 Iriszorg, Arnhem, The Netherlands; Centre for Addiction and Mental Health, CANADA

## Abstract

**Background:**

Attentional bias for substance-relevant cues has been found to contribute to the persistence of addiction. Attentional bias modification (ABM) interventions might, therefore, increase positive treatment outcome and reduce relapse rates. The current study investigated the effectiveness of a newly developed home-delivered, multi-session, internet-based ABM intervention, the Bouncing Image Training Task (BITT), as an add-on to treatment as usual (TAU).

**Methods:**

Participants (*N* = 169), diagnosed with alcohol or cannabis use disorder, were randomly assigned to one of two conditions: the experimental ABM group (50%; TAU+*ABM*); or the control group (50%; split in two subgroups the TAU+placebo group and TAU-only group, 25% each). Participants completed baseline, post-test, and 6 and 12 months follow-up measures of substance use and craving allowing to assess long-term treatment success and relapse rates. In addition, attentional bias (both engagement and disengagement), as well as secondary physical and psychological complaints (depression, anxiety, and stress) were assessed.

**Results:**

No significant differences were found between conditions with regard to substance use, craving, relapse rates, attentional bias, or physical and psychological complaints.

**Conclusions:**

The findings may reflect unsuccessful modification of attentional bias, the BITT not targeting the relevant process (engagement vs. disengagement bias), or may relate to the diverse treatment goals of the current sample (i.e., moderation or abstinence). The current findings provide no support for the efficacy of this ABM approach as an add-on to TAU in alcohol or cannabis use disorder. Future studies need to delineate the role of engagement and disengagement bias in the persistence of addiction, and the role of treatment goal in the effectiveness of ABM interventions.

## Introduction

The persistent nature of substance use disorders is well known among researchers and professionals working with addicted patients [1, 2]. On the basis of current dual process models it has been argued that the difficulty of controlling substance use behavior can be explained in part by an interplay of relatively automatic and controlled processes [3, 4]. Attentional Bias (AB) represents a category of relatively automatic processes that has been proposed to be related to the development and persistence of addictive behaviors. AB is defined as heightened attentional capture of substance-relevant cues and can be characterized by an initial direction of attention towards substance-relevant cues (i.e., engagement bias) and/or increased difficulty to disengage attention from these cues (i.e., disengagement bias; [5, 6]). Both components of AB may contribute to increased salience of substance-relevant cues, which in turn may contribute to the development of craving, thereby lowering the threshold for actual substance use (e.g., [7]). Accordingly, people may enter a self-reinforcing bias-craving-bias cycle that might gear them to repeated use of addictive substances [8]. Consistent with this possibility, it has been found that AB towards substance-relevant cues has been related to the severity of addiction and the probability of relapse after treatment ([9, 10]; but see [11] for a critical appraisal of the existing evidence).

To the extent that AB plays a role in the persistence of substance use behavior, it seems relevant to develop interventions to address AB. Importantly, recent studies found that AB is largely unaffected by current treatments that target deliberate (“reflective”) processes involved in decision-making and behavioral control [12, 13]. Another category of interventions has therefore been developed with the specific goal of modifying reflexive processes such as AB towards substance-relevant cues, to determine whether this reduces clinically-relevant addictive behavior such as excessive consumption, craving, and relapse. However, recent reviews suggest that the efficacy of these Attentional Bias Modification (ABM) trainings are mixed [14–16]. Several factors have been identified which might explain these inconsistent findings regarding ABM trainings and their efficacy on substance use disorder symptoms.

One potentially important factor that may limit the efficacy of ABM trainings concerns the methodology of typical ABM training procedures, which typically involve the presentation of just two static stimuli. For example, visual probe AB training, which is based on the visual probe task [17], requires participants to identify the identity of a probe that appears in the same position as a previously presented neutral, substance-irrelevant stimulus. This neutral stimulus is consistently positioned distally from a substance-relevant stimulus. The intention of presenting the probe in the position of the neutral stimulus is to train participants’ attention away from the substance-relevant stimulus [18]. The same task configuration holds for the Alcohol/Drug Attention Control Training Program, in which participants are instructed to repeatedly identify the colored outline of a substance-irrelevant stimulus while not attending to the simultaneously presented substance-relevant stimulus [19]. A limitation of these types of trainings is that they lack the complexity of real-life substance use situations, in which individuals are constantly surrounded by multiple stimuli, that are often dynamic in nature. This might limit the generalization from training effects to actual behavior. In addition, there are indications that the confined configuration of these trainings may not constitute a relevant challenge to the attentional system. Current ABM trainings might therefore be able to modify AB, but their capacity to impact real-life substance use behavior might be limited by their confined stimulus configuration [20].

A second potential factor contributing to the inconsistent findings of ABM trainings concerns variation in the number of training sessions. That is, several studies have shown that a single session of ABM training can modify AB [21, 22]. However, multiple sessions of ABM training seem necessary for modified AB to transfer into positive changes of substance use symptoms (e.g., [10, 23]). In addition, there are indications that multiple sessions of ABM training are also necessary to achieve a long-lasting modification of AB [24].

A third potential reason for inconsistency has been suggested based on results in anxiety-related ABM studies [25]. This concerns the context in which ABM trainings are delivered, and reflects the idea that the extent to which AB is expressed might vary across environments. Translating the findings of anxiety-related studies to the field of addiction, it seems reasonable to assume that AB for substance-relevant information might play a more pronounced role in familiar, craving-provoking environments in which individuals usually consume the substance, rather than in novel and substance unrelated environments such as a clinical setting [26]. From this perspective, ABM trainings might be more effective when delivered in a substance use-relevant context, such as the home environment, than in a substance use-irrelevant context, such as the clinical or lab setting.

A fourth potential factor that may contribute to variability of findings concerns participant motivation to change substance use behavior. That is, changes of behavior may be less likely to be observed when individuals are not motivated to change [27, 28]. It seems essential, therefore, to study transference effects of modified AB into concurrent symptom changes in clinical samples. However, previous ABM studies in addiction have mainly included non-clinical samples consisting of individuals who have no or limited motivation to change their substance use behavior [16]. A recent (Bayesian) meta-analysis that only included clinical studies, found a small effect of cognitive bias modification (combining both ABM and approach-bias modification) on bias-change and relapse but not on reductions in substance use [29].

Recognition that these various factors could collectively contribute to observed inconsistency in past findings suggests ways of potentially improving ABM training approaches, with the aim of increasing the clinically relevant effects of these interventions on substance use disorder symptoms. The current study was therefore designed to test a novel training that addresses the factors mentioned above, including (a) a more complex task configuration to more closely mimic real-life complexity, (b) multiple training sessions, (c) the delivery within the home environment, and (d) the inclusion of a treatment-seeking clinical sample.

Although this novel training addresses some of the issues raised regarding to the above-mentioned factors, there is an important challenge to multi-session trainings and the delivery of interventions at home, namely motivation. There are indications from the field of anxiety research that compliance can be limited when participants are required to complete multi-session trainings, at least in non-clinical samples (e.g., [30]). One potential reason for this is that participants report such trainings to be boring [31, 32]. To enhance motivation and to make the training more appealing, gamification elements were added to the current ABM training. It also has been suggested that adding motivational treatment components, such as motivational interviewing, might increase compliance with ABM trainings [33]. Therefore, the current study provided the ABM training as an add-on to treatment as usual (TAU; i.e., CBT-based intervention), in which therapists used motivational interviewing techniques as part of the standard treatment protocol to increase motivation to change, and to encourage the patients to perform the ABM training on a regular basis.

Thus far promising positive effects of cognitive bias modification approaches, and of ABM in particular, have been found when bias modification trainings has been delivered as part of patients’ inpatient treatment [34–36]. Therefore, it remains unclear whether similar effects can be found in patients who are treated in an outpatient setting, as is common for addiction treatment in the Netherlands where the current study took place. One study that included both inpatients and outpatients observed comparable positive effects to those obtained in studies which only included inpatients [10]. However, the sample of that study was rather small and there was insufficient power to test any possible differences between the two subsamples. Consequently, it remains of pressing importance to determine whether similar effects of ABM can be found in patients who are treated in an outpatient treatment setting. Therefore, the current sample consisted of outpatients diagnosed with alcohol or cannabis use disorder.

In summary, the current study was designed as a multi-center randomized controlled two-armed trial to investigate the efficacy of a novel ABM intervention in reducing clinically-relevant symptoms of substance use behavior. The ABM intervention was provided as a home-delivered multi-session training to alcohol and cannabis dependent outpatients as an add-on to TAU.

## Method

### Trial design

**Fig 1:**
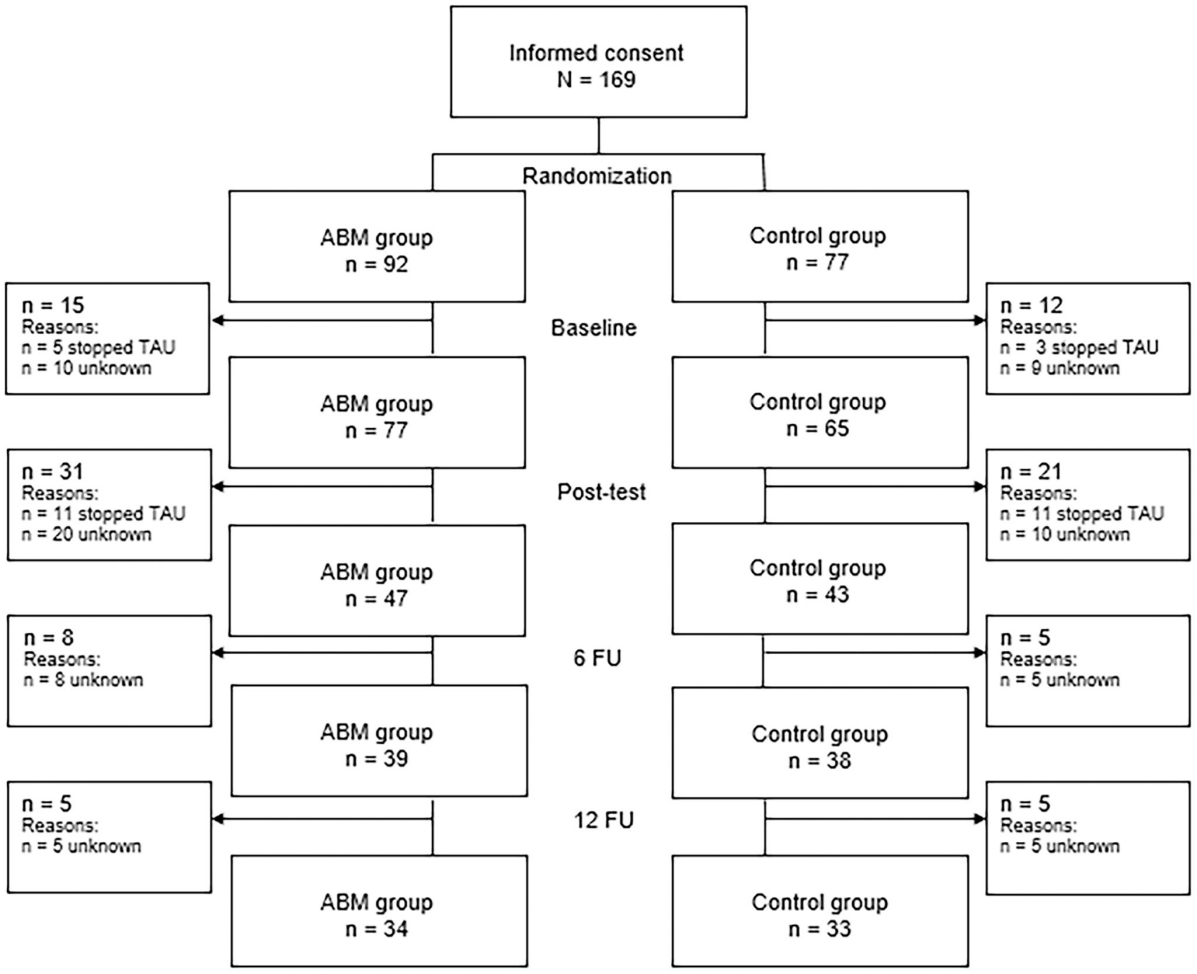
Participants flow-chart.

The present study was a multicenter randomized controlled two-armed, parallel-designed trial with one treatment arm (ABM intervention) and a control arm (see Fig 1). The design and procedure of the current study has been described in detail in the published study protocol [37].

### Participants

Patients were eligible for the study if they (a) were 18 years or older, (b) had a primary diagnosis of AUD or CUD, and (c) had an indication for and received TAU as described above. Patients were not eligible if they had a problem with gaming, gambling disorder, or internet addiction as measured with a short version of the C-VAT 2.0 (*n* = 1; [38]), and/or if they had no computer/laptop or no access to the internet (*n* = 2). Participants were treatment-seeking adult patients (73.4% male) diagnosed with alcohol use disorder (AUD; 74.6%) or cannabis use disorder (CUD; 25.4%), based on the diagnostic criteria of the Diagnostic and Statistical Manual of Mental Disorders Fourth/Fifth Edition [39], with a mean age of 43.20 (*SD* = 13.96; age range 18–78). Participants received TAU in line with current treatment policy and guidelines in Dutch addiction care, which consisted of 350 to 750 minutes of protocolized outpatient CBT-based intervention. The treatment goal of participants was either moderation or abstinence, depending on their own capacity and wishes, and the recommendation of the therapists. Participants experienced no or only limited secondary problems, such as financial or relational problems. See Fig 1 for a flow-chart indicating the drop-out of participants throughout the study. For most participants, the reason why they dropped-out of the study was unknown as the researchers could no longer could get in contact with them. After randomization and after baseline, there were several participants who dropped-out of the study because they (also) stopped TAU.

### Recruitment

Eligible patients of the involved treatment centers (Verslavingszorg Noord Nederland, Novadic-Kentron, Iriszorg, and Tactus) received information about the study from their therapist (i.e., a folder including a detailed description of the study and an informed consent form). Patients provided written permission for their contact information to be passed to the researcher who contacted the patients by phone to screen for eligibility, to explain the study, and to answer questions.

### Procedure

Approval for the current study was given by the ethical committee of the University Medical Centre of Groningen (UMCG; METc 2016/026), and the study was registered at the Netherlands Trial Register (NTR5497). Data collection took place between April 2016 and June 2019. This period was longer than originally planned and indicated in the pre-registration, which is due to slower inclusion of participants. This extension was approved by the grant agency (i.e., ZonMw).

**Fig 2:**
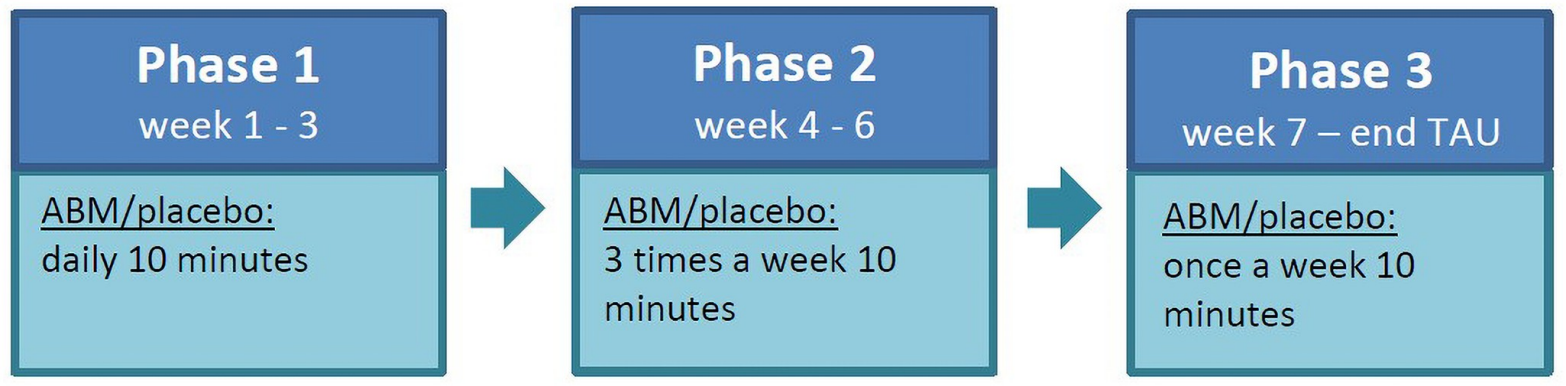
Training protocol.

Eligible patients who were willing to participate signed the informed consent form and sent it to the researcher. Afterwards they were assigned to the online registration and monitoring tool where they were automatically randomly allocated to one of the two conditions: ABM group (50%; TAU+*ABM*); control group (50%; TAU+*placebo* and TAU-only, 25% each; for details about randomization and blinding see below). After randomization, participants received an automated e-mail in which they were invited to start the baseline assessment. In order to prevent that potentially early effects of TAU would affect the baseline assessment, participants were requested to finish the baseline assessment before the fourth session of TAU. Patients who did not meet these requirements were excluded from further participation (*n* = 27). After baseline assessment, participants who were assigned to the ABM condition or to the placebo subgroup read the training instructions, and watched a short instruction video, followed by a five minutes practice session. For the subsequent three weeks, participants of the ABM condition and the placebo subgroup were invited to complete a training session on a daily basis. After this period, participants were invited to train for another three weeks three times a week, and thereafter once a week for the remaining time of TAU (see Fig 2). The exact number of (active or placebo) training invitations was, therefore, dependent on the duration of TAU, and the last invitation was sent once the therapists indicated the end of TAU. If participants missed three ABM/placebo sessions in a row, they received an automatic reminder via e-mail. If such participants did not resume the (active or placebo) training, a researcher contacted them to find out whether there were any practical problems, and to address any issues or concerns the participant may have, in the hope that this may enable the participant to continue regular engagement with the (active or placebo) training. Of course, participants were ultimately free to discontinue their involvement in the study should they choose to do so.

During TAU, the therapists and participants of the ABM condition and the placebo subgroup identified the time of the day when craving for the substance was strongest. The therapists then instructed their patients to train at this particular time of the day. Thereafter, during the regular therapy sessions, the therapists and their patients discussed the progress of the training, and if necessary, the therapists motivated the participants to train on a regular basis.

At the end of TAU, all participants received an automated invitation for the post-test assessment. Finally, 6 and 12 months later, all participants received another automated invitation for this two follow-up assessments (i.e., 6 FU, 12 FU). If a participants did not respond to the invitations and the automatic reminders, a researcher contacted them by phone to remind them to fill in the online measurements.

### Material

#### Intervention and placebo condition

**Fig 3:**
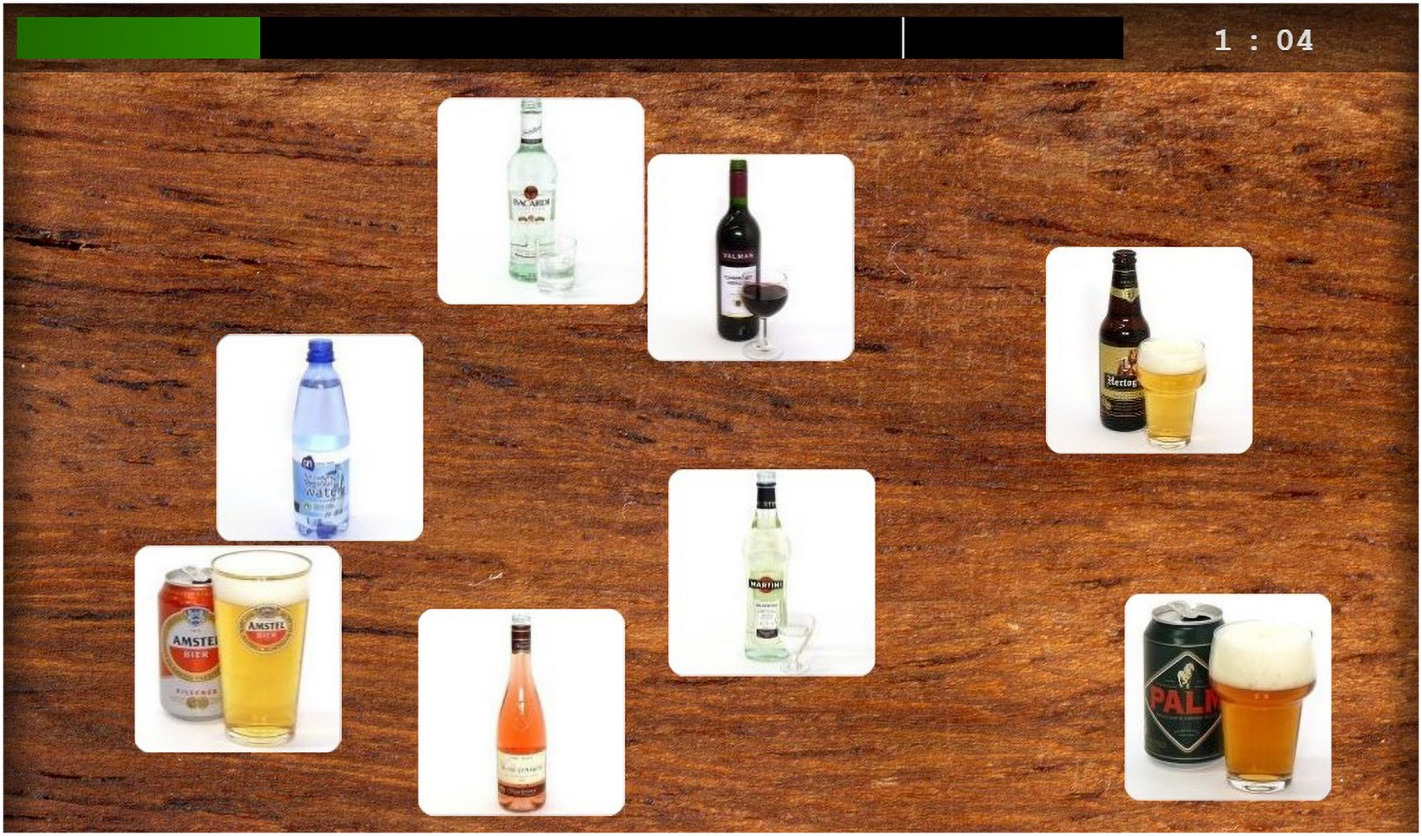
Screenshot of the alcohol version of the Bouncing Image Training Task.

*Attentional bias modification training*. AB for alcohol/cannabis cues was trained away with the Bouncing Image Training Task (BITT), based on the *Emotion-in-Motion* training [40]. In this computerized task, eight bouncing squares containing images are shown on a screen. Participants were instructed to follow the single substance-irrelevant image attentively with the mouse cursor, while ignoring the seven substance-relevant images (see Fig 3). Once the mouse cursor was on top of the substance-irrelevant image it briefly became green (500 ms), to show the participants that they were following the right image. At frequent unpredictable time intervals all images changed into other images. The substance-irrelevant image changed into (1) another substance-irrelevant image, or (2) a substance-relevant image. In case of the second possibility, participants were instructed to disengage from the substance-relevant image which appeared in the square and to find the new substance-irrelevant image as quickly as possible.

Each training session was divided into four games of 2.5 minutes (10 minutes in total), and the training consisted of 12 levels, gradually increasing in difficulty. All participants started with level one and could unlock more challenging levels by reaching 80 points or more (with a maximum score of 100). The points were calculated based on the amount of time participants were tracking the substance-irrelevant image. For each level the high score was stated, so that participants could challenge themselves during the next game to reach a higher score. During each training block, participants were able to track their progress in a green bar shown on the screen. These gamifications were included to enhance motivation and to make the training more appealing.

*Placebo condition*. The placebo condition was designed to be similar to the active training, meaning that the stimuli, the design/layout, the temporal parameters, and the construction of levels were equal to the BITT. Thereby the placebo condition was suited to account for possible exposure effects of the alcohol/cannabis cues, and effects of adding a component to TAU. As the placebo condition was not configured to change attentional patterns towards substance-relevant cues, four squares containing substance-relevant images and four squares containing substance-irrelevant images moved on the screen. Participants were instructed to pay equal attention to all eight moving squares, and on random occasions, one of the images became green-filtered. Participants needed to click on this green-filtered image as quickly as possible. Both types of images (i.e., substance-relevant and substance-irrelevant) became green-filtered equally often (50:50).

*Stimuli*. There were two different task variants for the BITT as well as for the placebo condition–one alcohol and one cannabis version. For each of these two variants two sets of 64 images (500 x 500 pixel) were assembled. The first set of images for both variants was used during all of the training sessions, except for the last session in which the second set of images was used to measure generalization to untrained stimuli. This last set of images was activated by a researcher once the therapists indicated the end of TAU. For the alcohol variant, each of these two sets of images compromised 32 alcohol-relevant images (i.e., alcoholic drinks), and 32 alcohol-irrelevant images (i.e., non-alcoholic drinks). For the cannabis variant, each of the two sets of images consisted of 32 cannabis-relevant images (i.e., objects related to cannabis use), and 32 cannabis-irrelevant images (i.e., office supplies; [12, 41]).

For each of the four games per session of the alcohol and cannabis variant of the BITT and placebo condition, eight different substance-relevant images and eight different substance-irrelevant images were randomly drawn from the activated set of 64 images, meaning that all 64 images of the first set of images were presented to the participants within each training session, except for the last training session in which all 64 images of the second set of images were presented.

#### Behavioral measures

AB (i.e., engagement bias and disengagement bias) was measured with the Odd-One-Out assessment task (OOOT; [42]). In this task participants had to correctly indicate whether or not an odd-one-out image (i.e. a single image that did not belong to the same category as all other images) was present in a 4 x 5 matrix of 20 images. Participants had a maximum of 10 seconds on each trial to respond by pressing the ‘0’ (i.e., no odd-one-out present) or ‘1’ (i.e., odd-one-out present) button of the keyboard. Each trial started with the presentation of a red fixation cross in the middle of the screen (500 ms).

In line with the intervention and placebo condition, there was an alcohol and a cannabis variant of the Odd-One-Out assessment task. Each of these variants used three types of stimulus images, and each of these three stimulus sets comprised of 30 images (total 90 images per task variant), which were all different from the stimuli that were used for the BITT and placebo condition. For the alcohol variant of the assessment task, the three images types were alcoholic drinks, non-alcoholic-drinks, and flowerpots, whereas for the cannabis variant the three images types were cannabis-related objects, neutral daily devices, and flowers. The images of the flowerpots of the alcohol variant and the images of flowers of the cannabis variant were selected for the purpose of the current study. The other images were used in previous studies [12, 41]. Given that there were three different categories of images for each variant, nine different combinations of trials were possible (see Table 1), including three conditions without an odd-one-out, and six different odd-one-out combinations. The data of interest to calculate AB were the trials including an odd-one-out. The latencies of correct responses on these trials were processed. Both variants of the task were divided into three blocks of 24 trials each (72 trials in total). The order of trials was random, and the odd-one-out image randomly appeared over the possible positions, but never directly above or below the fixation cross.

**Table 1:** Type and number of trials in the alcohol and cannabis Odd-One-Out Task (OOOT).

		Trial types alcohol OOOT		Trial types cannabis OOOT		Trials per game
**No odd-one-out trials**	**1**	Alcoholic drinks (20)		Cannabis-related objects (20)		2
	**2**	Non-alcoholic drinks (20)		Neutral daily devices (20)		2
	**3**	Flowerpots (20)		Flowers (20)		2
		**Target**	**Distractors**	**Target**	**Distractors**	
**Odd-one-out trials**	**4**	Alcoholic drinks (1)	Non-alcoholic drinks (19)	Cannabis-related objects (1)	Neutral daily device (19)	3
	**5**	Alcoholic drinks (1)	Flowerpots (19)	Cannabis-related objects (1)	Flowers (19)	3
	**6**	Non-alcoholic drinks (1)	Alcoholic drinks (19)	Neutral daily device (1)	Cannabis-related objects (19)	3
	**7**	Flowerpots (1)	Alcoholic drinks (19)	Flowers (1)	Cannabis-related objects (19)	3
	**8**	Non-alcoholic drinks (1)	Flowerpots (19)	Neutral daily device (1)	Flowers (19)	3
	**9**	Flowerpots (1)	Non-alcoholic drinks (19)	Flowers (1)	Neutral daily device (19)	3

*Note*. Number of presented images per trial is given between the parentheses. Trial numbers 4 and 5 (i.e., alcohol target trials; cannabis target trails), trial numbers 6 and 7 (i.e., alcohol distractors trials; cannabis distractors trials) and trial numbers 8 and 9 (i.e., neutral target in neutral distractors trials) were included in the current analyses.

For the alcohol variant of the task, engagement bias was calculated by subtracting the mean reaction time of the alcohol target trials (i.e., 1 alcohol image among 19 non-alcoholic drinks images or 19 flowerpot images) from the mean reaction time of the neutral target in neutral distractors trials (i.e., 1 non-alcoholic drink image among 19 flowerpot images or 1 flowerpot image among 19 non-alcoholic drinks images). For the cannabis variant, engagement bias was calculated by subtracting the mean reaction time of the cannabis target trials (i.e., 1 cannabis image among 19 daily devices images or 19 flower images) from the mean reaction time of the neutral target in neutral distractors trials (i.e., 1 daily device image among 19 flower images or 1 flower image among 19 daily device images). Higher scores thus reflected stronger attentional engagement with alcohol or cannabis cues. Disengagement bias for the alcohol variant was calculated by subtracting the mean reaction time of the neutral target in neutral distractors trials from the mean reaction time of the alcohol distractors trials (i.e., 1 non-alcoholic drink image or 1 flowerpot image among 19 alcohol images). For the cannabis variant, disengagement bias was calculated by subtracting the mean reaction time of the neutral target in neutral distractors trials from the cannabis mean reaction time of the cannabis distractors trials (i.e., 1 daily device image or 1 flower image among 19 cannabis images). Higher scores reflected stronger difficulty to disengage from alcohol or cannabis cues.

#### Self-report measures

*Substance use*, *craving*, *and depression*, *anxiety and stress*. The frequency of alcohol/cannabis use, the number of standard units of alcohol consumed (for AUD only), craving, and depression, anxiety and stress levels were measured using the relevant parts of the Measurements in Addiction of Triage and Evaluation Questionnaire (MATE-Q; [43]). The frequency of alcohol use was assessed by asking participants on how many days of the past 30 days they consumed alcohol. In addition, they indicated how many standard glasses of alcohol they drank on a regular drinking day. For cannabis, the participants indicated on how many days of the past 30 days they used weed/hash. Craving was assessed with an abbreviated version of the Obsessive-Compulsive Drinking Scale (OCDS) of the MATE-Q. This scale consists of five items measuring the desire for alcohol/cannabis in the past seven days. The Depression Anxiety Stress Scale (DASS) of the MATE-Q was used to assess participants’ amount of depressive and anxious feelings, and their stress level. The MATE-Q was assessed in the form of an interview together with the therapist during the intake and the last session of TAU. At 6 FU and 12 FU, participants completed the questionnaire online. Separate items of the MATE-Q were only available for the internet-assessed questionnaires. Therefore, we were only able to calculate the reliability of the OCDS5 and the DASS for 6 FU and 12 FU. Reliability of the OCDS5 as estimated with Cronbach’s alpha was good at 6 FU (α = .83), and 12 FU (α = .84). For the DASS, Cronbach’s alpha was good for both 6 FU and 12 FU (α = .95; α = .96, respectively).

*Other measurements*. At baseline, sociodemographic information was collected, including gender, age, level of education, relationship, and work. In addition, participants’ clinical history of addiction, as well as their family history of addiction (first and second grade relatives) was assessed. At the end of the baseline assessment, all participants filled in a short questionnaire about their use of technical devices like computers and mobile phones. Participants who were assigned to the active or placebo training were asked about their expectations concerning the intervention on a 5-point Likert scale (ranging from ‘totally agree’ to ‘totally disagree’) after they completed a practice session. In addition, before and after each (active or placebo) training session participants were asked to indicate their level of subjective craving on a visual analogue scale (VAS), varying from 0 (“no craving”) to 100 (“extreme craving”). Direct effects of the training on craving could therefore be established. At 6 FU and 12 FU, participants were asked whether they started to use alcohol/cannabis again (i.e., for participants who had abstinence as their goal of treatment), or whether they started to use more alcohol/cannabis than they intended to (i.e., for participants who had moderation as the goal of their treatment). If participants answered the question with yes, they were asked to indicate when they have had experienced a relapse (i.e., number of months after the end of TAU). Finally, after the first week of training and at post-test, participants assigned to the (active or placebo) training filled in an evaluation form asking them about their training experiences as indicated on a VAS varying from 0 (not at all) to 100 (very much).

### Sample size

Based on power analysis, the current study aim was to include 213 participants, which affords the capacity to find a difference between groups of a medium effect size of Cohen’s *d* = 0.5, based on a *t*-test for independent groups, with a power of 0.8 and an alpha of 0.05, allowing for a dropout rate of 40%. However, due to political and organizational factors (e.g., reorganization, high workload of therapists) the recruitment of participants into the study was delayed. Despite the addition of a fourth treatment center and a series of intense efforts (e.g., to facilitate therapists) recruitment fell short of this target, and we were able to include a sample of 169 participants. With this sample the power to detect a medium effect size of Cohen’s *d* = 0.5 at an alpha of 0.05, when allowing a drop-out of 40%, was 0.7.

### Randomization and blinding

Eligible participants were automatically randomized by the computerized online registration and monitoring tool, called LOTUS. Randomization was stratified for gender, age group (18–30, 30–50, 50+), type of addiction, and institution, meaning that participants were automatically assigned to the condition to which the fewest participants of their gender, age group, and type of addiction were already assigned accounting for the institution in which the participants were treated. Randomized intervention assignment was concealed from both patients and therapists. Furthermore, since all assessments took place online, and thus in the absence of the therapists and researchers, the outcome data were blinded for both therapists and researchers. One researcher was aware of the allocation concealment to enable the support of participants in case any technical or personal problems occurred (for more detail see [37]).

### Analyses

The analyses were based on data of participants who at least completed the baseline assessment (*n* = 142), meaning that participants who provided informed consent but stopped being involved before completing baseline assessment (*n* = 27) were considered as drop outs. To analyze the data of the included participants over all four measurements, missing data were handled with multiple imputation. The percentage of missing values for the primary outcome variable frequency of use was 29.6% at post-test, 61.3% at 6 FU, and 52.8% at 12 FU. For craving, the percentage of missing values was 51.4% at post-test (higher values than for the frequency of use because for some participants craving was not assessed by the therapist during the final session of TAU), 61.3% at 6 FU, and 52.8% at 12 FU. A multiple imputation model was constructed using the *R* package *mice* (multiple imputation by chained equations, version 3.6.0; [44]) in which variables were either used as predictor (to impute other variables) and imputed themselves, or only used as predictor. To keep the model feasible, variables that were expected to have no direct influence on the primary outcome measures were excluded from the imputation model (e.g., nationality and relationship). Moreover, cross-time interactions between different variables were not allowed in the imputation model (e.g., baseline alcohol use was not used to impute post-test craving; see S1 and S2 Appendices for the specification of the imputation model). Based on the percentage of missing values at 12 FU, the incomplete dataset was imputed 50 times. The obtained imputed datasets were exported to IBM SPSS Statistics for Windows (version 25.0; [45]). In SPSS, following an intention to treat approach, changes in engagement bias and disengagement bias (i.e., manipulation check), as measured with the OOOT, were investigated by conducting two 4 (within subjects: baseline, post-test, FU 6, FU 12) × 2 (between subjects: ABM versus control condition) Repeated Measures Analyses of Variance (RM-ANOVAs). To test the main hypotheses, two 4 (within subjects: baseline, post-test, FU 6, FU 12) × 2 (between subjects: ABM versus control condition) RM-ANOVAs were conducted to investigate the effects of the ABM intervention on the frequency of used substance and craving as measured with the MATE-Q. Finally, differential changes in secondary physical and psychological complaints (depression, anxiety, and stress) were examined by a 4 (within subjects: baseline, post-test, FU 6, FU 12) × 2 (between subjects: ABM versus control condition) RM-ANOVA, based on the MATE-Q. After completing the analyses in SPSS, the results were read into *R* for the pooling of the F-values from the RM-ANOVAs following the procedure as described by van Buuren [46].

Based on the available data, internal consistency of the OOOT measures at baseline, post-test, 6 FU, and 12 FU was evaluated by using the split-half method to calculate Spearman-Brown coefficients between the first half and the second half of the task. A second method was used to account for a possible learning effect throughout the task. Therefore, Spearman-Brown coefficient was also calculated by distributing the trials alternately to one of two subsets, whereas the first trial of one particular trial type was randomly allocated to either of the subsets. Internal consistency was tested for engagement and disengagement bias, as well as for the trial types. The estimates for the internal consistency were characterized as weak (*r* < .5), adequate (.5 ≤ *r* < .8), or good (*r* ≥ .8) based on commonly reported thresholds [47].

## Results

### Data reduction OOOT

The approach of data reduction followed established convention adopted in previous research (see for example [48]). The same steps were followed for all four assessments (i.e., baseline, post-test, 6 FU and 12 FU). As low accuracy on the OOOT might indicate non-serious participation, participants scoring 3 *SD*’s below the mean percentage correct answers were removed (baseline *n* = 2). As a next step, incorrect responses were excluded from the analyses (baseline 37.6%; post-test 32.2%; 6 FU 27.7%; 12 FU 26.9%). Reaction times below 200 ms were considered anticipation errors and were removed from the analyses (baseline 4 trials; post-test 53 trials; 6 FU 8 trials; 12 FU 21 trials). Trials scoring 3 *SD*’s below or above a participant’s average response time of that trial type were removed. This resulted in deleting another 0.5% of trials from the baseline, 0.5% from the post-test, 0.3% from the 6 FU, and 0.4% from the 12 FU. See Table 2 for the internal consistency of the engagement and disengagement bias, as well as for all the trial types for baseline, post-test, 6 FU, and 12 FU.

**Table 2:** Internal consistency of the AB indices and all trial types of the OOOT at baseline, post-test, 6 months and 12 months FU.

		Baseline	Post-test	6 FU	12 FU
Engagement index	*Split half*	.37	-.21	.02	.45
	*Split half random*	.36	.18	.67	.21
Disengagement index	*Split half*	.29	.31	.37	.66
	*Split half random*	.46	.45	.67	.58
Target trials	*Split half*	.83	.84	.80	.81
	*Split half random*	.82	.86	.88	.86
Distractors trials	*Split half*	.76	.91	.80	.72
	*Split haf random*	.88	.91	.84	.82
Neutral trials	*Split half*	.71	.80	.85	.59
	*Split half random*	.81	.84	.82	.62

*Note*. Internal consistency is given as Spearman-Brown coefficient; target trials = alcohol/cannabis target trials; distractors trials = alcohol/cannabis distractors trials; neutral trials = neutral target in neutral distractors trials; split half = internal consistency as derived from the split half method in which the first half and the second half of the task are compared; split half random = internal consistency as derived from distributing the trials alternately to one of two subsets, whereas the first trial of one particular trial type was randomly allocated to either of the subsets.

### Descriptives

Based on the original data, sociodemographic information of participants in the ABM group and in the control group are presented in Table 3. There were no significant differences between the participants in the ABM group and the control group with regard to age, gender, and substance use disorder. After completing the practice session, 53.3% of participants in the ABM group, and 60.6% in the placebo subgroup indicated to expect a positive influence of the training on their attention to substance-relevant cues. Further, 59.8% of the ABM group, and 57.6% of the placebo subgroup expected that the training would help them with moderation or abstinence. Finally, 63.3% of the ABM group, and 63.7% of the placebo subgroup expected the training to have a positive influence on their overall treatment outcome. On average, participants of the ABM group completed 11.94 (*SD* = 12.69; range 0–45) training sessions. Participants of the placebo subgroup completed 9.79 (*SD* = 10.38; range 0–36) sessions on average. On average, participants of the ABM group unlocked 8.60 of the 12 levels (*SD* = 3.89; range 1–12), whereas participants of the placebo subgroup unlocked 10.83 of the 12 levels (*SD* = 2.28; rage 4–12). Based on all available data of the training sessions, on average, level of subjective craving in the ABM group before the training was 21.92 (*SD* = 26.47), and 19.70 (*SD* = 25.25) after. In the placebo subgroup, level of subjective craving was 25.03 (*SD* = 28.80) before the training, and 23.97 (*SD* = 26.15) after completing the placebo condition.

**Table 3:** Descriptives of sociodemographic information per condition.

	ABM group (*n* = 77)	Control group (*n* = 65)
Age (m, SD)	44.51 (13.37)	44.58 (14.57)
Gender (% male)	71.4	73.8
Alcohol use disorder (%)	77.9	78.5
Cannabis use disorder (%)	22.1	21.5
Technical devices (more than 3; %)	61.1	67.7
Participants treatment history (previous treatment; %)	26.0	30.8
Family history of addiction (first grade; %)	50.9	43.1
Family history of addiction (second grade; %)	44.2	38.5
Education (completed upper secondary education; %)	95.4	95.4
Education (completed tertiary education; %)	53.3	60.0
Partner (%)	64.9	67.7
Work (study, employment, or self-employed; %)	66.2	70.8

*Note*. Age is indicated by means and standard deviations. All other descriptives are indicated in percentages.

In Tables 4 and 5, based on the imputed data, AB indices and mean reaction times for all three trial types (i.e., *target trials*, *distractors trials*, and *neutral target in neutral distractors trials*), as well as the mean frequency of substance use, craving, and secondary physical and psychological symptoms are presented per group for baseline, post-test, 6 FU, and 12 FU. No differences were found between the placebo subgroup and the TAU-only subgroup on the primary outcome variables frequency of use (post-test: *t* = -1.24, *p* = .216; 6 FU: *t* = -0.95, *p* = .341; 12 FU: *t* = -0.66, *p* = .510) and craving (post-test: *t* = -0.32, *p* = .749; 6 FU: *t* = -0.13, *p* = .897; 12 FU: *t* = -0.30, *p* = .763). This is consistent with the idea that the placebo training would have no effect on relevant symptoms of substance use disorders (see S3 Appendix for the means and standard deviations separated for the subgroups of the control condition). For participants who indicated to have had a relapse, the number of months until relapse (i.e., started using alcohol/cannabis or used more than intended, depending on the goal of treatment), as calculated based on the imputed data, was 3.24 (*SD* = 2.02) for the ABM group, and 2.99 (*SD* = 1.91) for the control group (*t* = -0.46, *p* = .644). The percentage of participants who reported no relapse was 38.4% in the ABM group, and 34.8% in the control group (χ^2^ (1046.33, *N* = 142) = 0.36, *p* = .550).

**Table 4:** Descriptives of the manipulation check per condition and time point.

	ABM group (*n* = 77)				Control group (*n* = 65)			
	*Baseline*	*Post-test*	*6 FU*	*12 FU*	*Baseline*	*Post-test*	*6 FU*	*12 FU*
Engagement index	-107 (641)	-367 (698)	-37 (1223)	-184 (794)	-158 (786)	-318 (685)	-435 (1265)	-270 (819)
Disengagement index	783 (970)	1071 (1013)	1045 (1257)	927 (1132)	582 (1035)	702 (939)	850 (1322)	940 (1231)
Target trials	3328 (1107)	2961 (1294)	2627 (1564)	2871 (1147)	3092 (850)	2988 (1211)	3001 (1581)	2720 (987)
Distractors trials	4011 (1389)	3675 (1743)	3607 (1599)	3621 (1495)	3516 (1144)	3407 (1442)	3360 (1640)	3407 (1427)
Neutral trials	3216 (1168)	2602 (1272)	2637 (1206)	2649 (975)	2939 (826)	2688 (1148)	2625 (1154)	2457 (840)

*Note*. Means and standard deviations of the AB indices and types of trials are given in ms; neutral trials = neutral target in neutral distractors trials; all standard deviations are presented in parenthesis.

**Table 5:** Descriptives of the primary outcome variables substance use and craving and the secondary outcome variable per condition and time point.

	ABM group (*n* = 77)				Control group (*n* = 65)			
	*Baseline*	*Post-test*	*6 FU*	*12 FU*	*Baseline*	*Post-test*	*6 FU*	*12 FU*
Frequency substance use	17.92 (11.49) [15.30; 20.54]	7.72 (11.03) [5.19; 10.25]	8.82 (10.58) [5.31; 12.31]	8.97 (10.66) [5.52; 12.42]	20.15 (11.56) [17.31; 22.99]	9.10 (10.31) [6.23; 11.98]	11.70 (11.70) [7.92; 15.45]	12.69 (12.11) [9.39; 15.99]
Craving	7.23 (3.44) [6.28; 8.17]	4.34 (4.40) [2.95; 5.73]	9.42 (4.03) [8.02; 10.83]	9.43 (4.51) [7.81; 11.05]	7.10 (4.20) [6.07; 8.12]	5.60 (4.90) [4.93; 7.26]	9.72 (4.43) [8.17; 11.28]	9.93 (4.51) [8.36; 11.50]
DASS	30.30 (22.97) [24.54; 36.06]	19.74 (21.32) [13.13; 26.36]	65.15 (20.27) [58.14; 72.15]	63.81 (20.82) [56.83; 70.79]	32.77 (21.14) [26.53; 39.01]	24.53 (22.69) [16.83; 32.24]	67.44 (20.23) [60.91; 73.96]	64.02 (20.52) [57.47; 70.58]

*Note*. All standard deviations are presented in parenthesis and all 95% confidence intervals are presented in square brackets.

Based on the available data from the ABM group (*n* = 36 after first week of training, *n* = 34 at post-test), and the placebo subgroup (*n* = 14 after first week of training, *n* = 15 at post-test), after the first week of training, the mean motivation to train regularly was 55.94 (*SD* = 24.73, range 6–100) in the ABM group, and 57.86 (*SD* = 27.90, range 0–100) in the placebo subgroup. On average, participants’ judgment about whether or not the training would be helpful with regard to their treatment outcome was 38.89 (*SD* = 25.99, range 0–92) for the ABM group, and 31.50 (*SD* = 21.71, range 6–65) for the placebo subgroup. At post-test, the extent of motivation to train on a regular basis throughout the treatment was 51.76 (*SD* = 29.03, range 5–100) in the ABM group, and 45.20 (*SD* = 26.74, range 4–79) in the placebo subgroup. The judgment about whether or not the training had a positive influence on their treatment outcome was on average 34.56 (*SD* = 31.09, range 0–100) in the ABM group, and 31.80 (*SD* = 23.18, range 2–81) in the placebo subgroup, which was comparable with the answers after the first week. Further, participants in the ABM group gave a mean pleasantness rating following the ABM intervention of 45.41 (*SD* = 27.14, range 3–100). For participants of the placebo subgroup this was 46.53 (*SD* = 20.68, range 20–71). On average, the fact that the intervention was completed from home was rated as positive (*M* = 85.68, *SD* = 16.79, range 30–100; *M* = 83.07, *SD* = 16.27, range 50–100, respectively for participants of the ABM group and placebo subgroup). Participant ratings concerning the frequency with which their therapist had asked about the training during TAU, varied from (almost) every session (20.5% for the ABM group and 20.0% for the placebo subgroup) to never (29.4% for the ABM group and 31.3% for the placebo subgroup).

### Impact of ABM procedure on AB, substance use, craving, and physical and psychological complaints

The assumption of sphericity as indicated by Mauchly’s tests was violated for most effects of all four RM-ANOVAs. Therefore, degrees of freedom were corrected using Greenhouse-Geisser estimates of sphericity. The RM-ANOVA testing whether the ABM intervention was successful in manipulating AB, revealed no significant main effect of time for engagement bias (*F*(2.38, 160.65) = 1.66, *p* = .187, *η*^*2*^*p =* 0.02), and disengagement bias (*F*(2.76, 205.14) = 1.98, *p* = .123, *η*^*2*^*p =* 0.03). Most important for the context of the current study, there was no interaction of time and condition for engagement bias (*F*(2.38, 121.34) = 0.96, *p* = .397, *η*^*2*^*p =* 0.02), nor for disengagement bias (*F*(2.76, 131.55) = 0.47, *p* = .689, *η*^*2*^*p =* 0.01). This indicates that the change over time in AB was not different between the ABM group and the control group. Effects of generalization to untrained stimuli could not be assessed as only a very small number of participants completed the last training session before the end of TAU.

**Fig 4:**
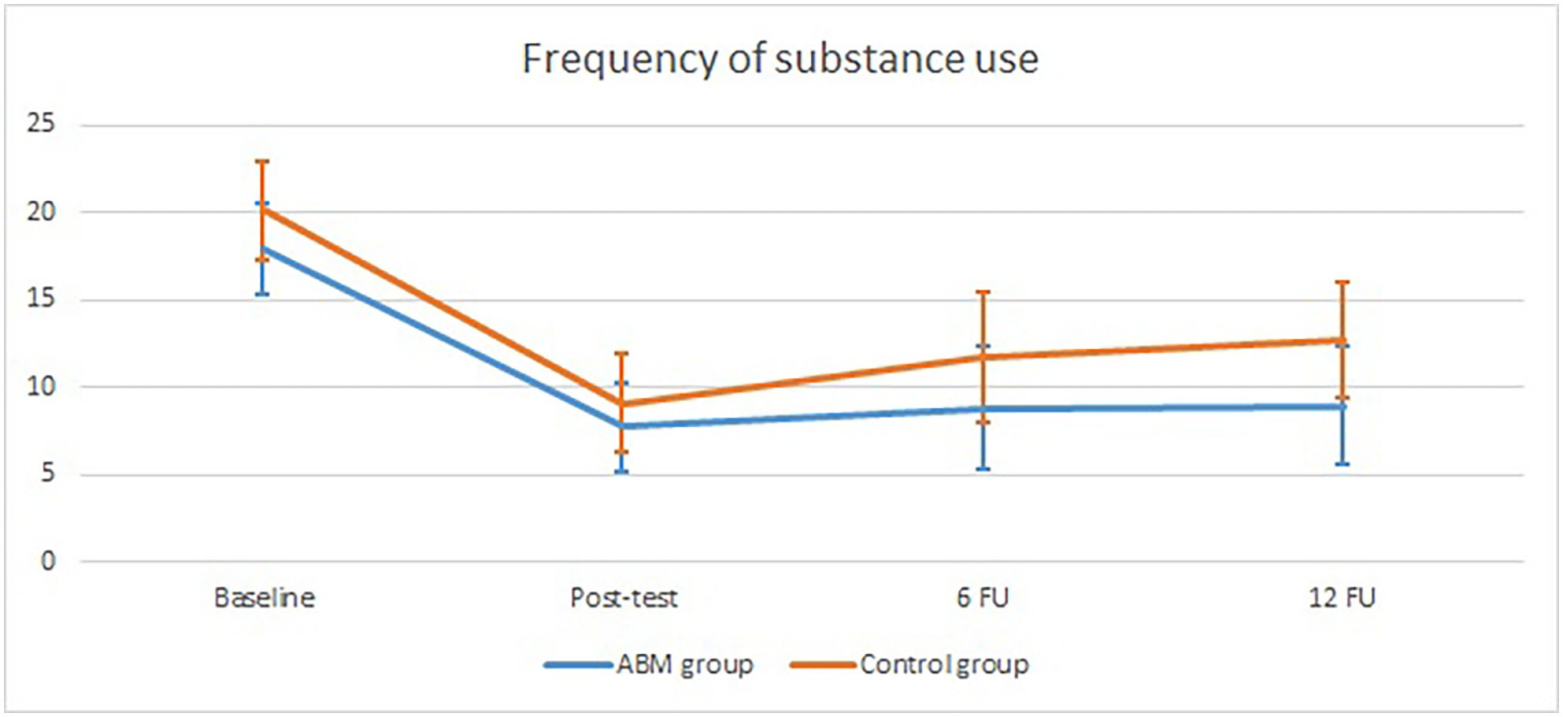
Frequency of used substance in the past 30 days for the ABM group and the control group at baseline, post-test, 6 FU, and 12 FU.

With regard to the frequency of consumed substance, there was a significant main effect of time, *F*(2.61, 175.38) = 28.75, *p* < .001, *η*^*2*^*p* = 0.27. Repeated contrasts and means revealed that overall there was a significant decrease of the frequency of substance use from baseline to post-test *F*(1, 461.43) = 92.01, *p* < .001, *η*^*2*^*p* = 0.46 (*M*_baseline_ = 18.94, *SD* = 11.54; *M*_post-test_ = 8.35, *SD* = 10.70), but no significant change from post-test to 6 FU, *F*(1, 59.91) = 2.12, *p* = .151, *η*^*2*^*p* = 0.04, and from 6 FU to 12 FU, *F*(1, 115.06) = 0.74, *p* = .391, *η*^*2*^*p* = 0.01. Further, there was no significant interaction effect between time and condition, *F*(2.62, 348.83) = 0.46, *p* = .685, *η*^*2*^*p* = 0.01, indicating that over time the frequency of substance use showed a similar pattern for the ABM group and the control group (see Fig 4).

**Fig 5:**
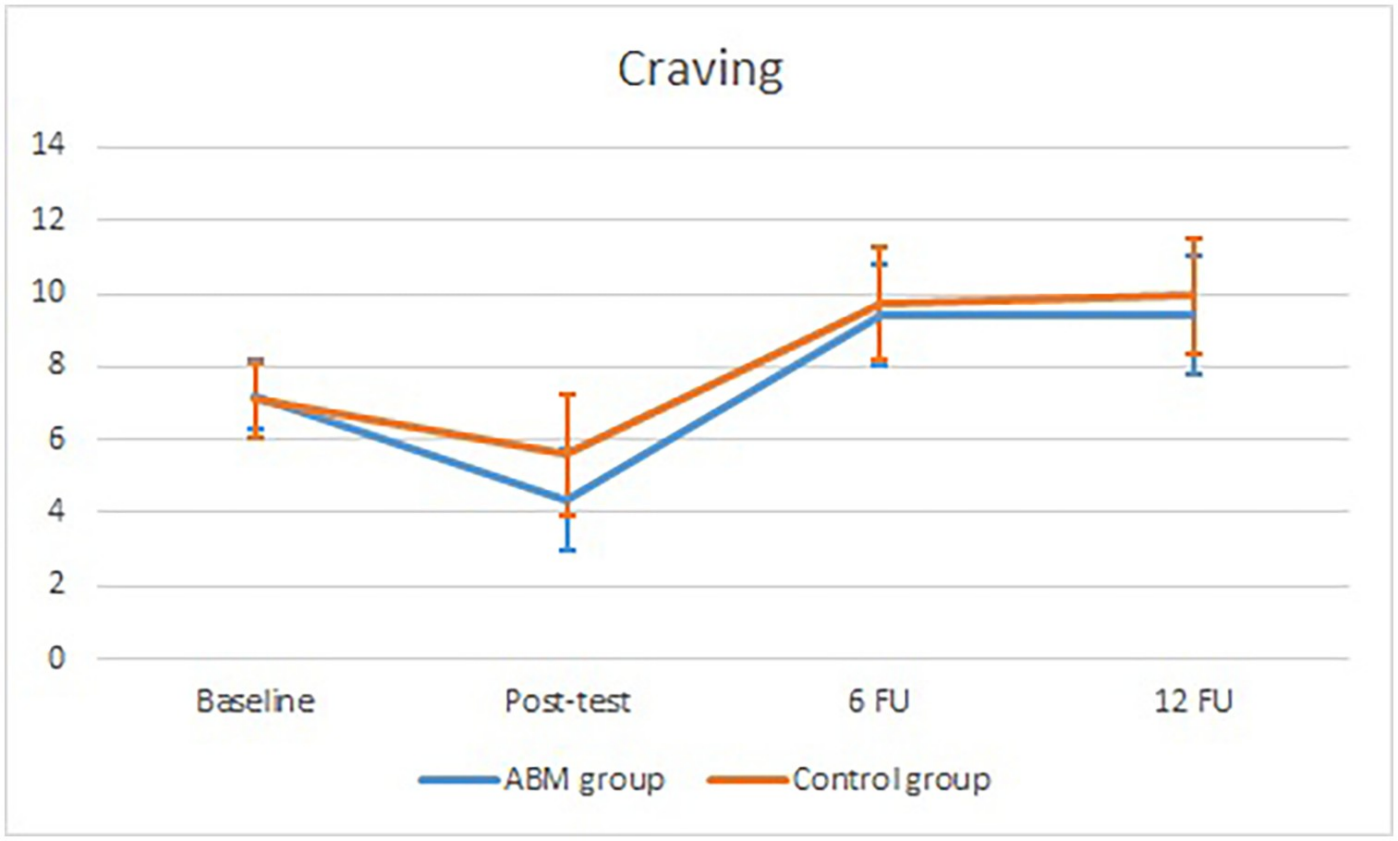
Craving for the ABM group and the control group at baseline, post-test, 6 FU, and 12 FU.

Similarly, there was a significant main effect of time for craving, *F*(2.70, 66.20) = 22.49, *p* = .001, *η*^*2*^*p* = 0.33. As indicated by the repeated contrasts and means, overall craving decreased significantly from baseline to post-test, *F*(1,69.70) = 17.25, *p* = .001, *η*^*2*^*p* = 0.21 (*M*_baseline_ = 7.17, *SD* = 3.80; *M*_post-test_ = 4.91, *SD* = 4.66), and showed a significant increase from post-test to 6 FU, *F*(1,37.12) = 36.41, *p* < .001, *η*^*2*^*p* = 0.46 (*M*_post-tst_ = 4.91, *SD* = 4.66; *M*_6FU_ = 9.56, *SD* = 4.22). The change from 6 FU to 12 FU was non-significant, *F*(1,120.76) = 0.87, *p* = .353, *η*^*2*^*p* = 0.01. The interaction effect between time and condition was non-significant, *F*(2.70,134.16) = 0.75, *p* = .510, *η*^*2*^*p* = 0.02, indicating that the development of craving over time was similar for participants in both groups (see Fig 5).

For the secondary outcome measure, secondary physical and psychological complaints, there was a significant main effect of time, *F*(2.36, 42.32) = 79.35, *p* < .001, *η*^*2*^*p* = 0.70. Contrasts and means revealed that secondary physical and psychological complaints decreased from baseline to post-test, *F*(1, 70.87) = 11.55, *p* = .001, *η*^*2*^*p* = 0.15, and significantly increased from post-test to 6 FU, *F*(1, 19.03) = 86.46, *p* < .001, *η*^*2*^*p* = 0.77. There was no significant change from 6 FU to 12 FU, *F*(1, 34.05) = 1.22, *p* = .278, *η*^*2*^*p* = 0.03. There was no significant interaction between time and condition, *F*(2.36, 157.94) = 0.57, *p* = .594, *η*^*2*^*p* = 0.01. This indicated that changes of symptoms of depression, anxiety, and stress over time did not differ between groups.

### Post-hoc exploratory analyses

To test whether the ABM training had direct effects on AB, two additional RM-ANOVAs were conducted in which possible direct changes from baseline to post-test for engagement and disengagement bias were investigated. However, the interaction term of time by condition remained non-significant (*F*(1, 270.17) = 0.60, *p* = .440; *F*(1, 142.31) = 0.63, *p* = .427, for engagement bias and disengagement bias, respectively), indicating that both groups showed the same pattern over time. To further analyze possible effects of the ABM intervention on the primary outcome variables (i.e., substance use and craving), we conducted several post-hoc RM-ANOVAs. First, we tested the effects of ABM intervention on substance use and craving when only including patients who completed a substantial number of (active or placebo) training sessions, namely at least six (see for example [35]). However, the interaction term of time by condition remained non-significant (*F*(2.58, 1133.90) = 0.62, *p* = .576; *F*(2.72, 172.45) = 0.43, *p* = .711, respectively for frequency of substance use and craving). In line, there were no significant differences concerning baseline frequency of substance use and craving between participants who completed a maximum of one session (*M*_frequency_ = 20.67, *SD* = 10.11; *M*_craving_ = 7.38, *SD* = 3.22) compared with participants who completed at least six sessions (*M*_frequency_ = 16.06, *SD* = 11.96; *M*_craving_ = 7.18, *SD* = 3.43). Second, when adding the type of used substance (i.e., alcohol or cannabis) to the model as a between-subjects factor, in order to investigate possible differences of the effect of ABM intervention between AUD and CUD, we found no significant three-way interaction between time, condition and type of substance (*F*(2.61, 340.25) = 0.46, *p* = .681; *F*(2.69, 168.43) = 0.65, *p* = .570, respectively for frequency of substance use and craving). Third, we investigated whether there was a difference between groups over time when separately including the subgroups of the control condition (i.e., placebo subgroup and TAU-only subgroup) into the model. However, there was no significant interaction of time by condition for frequency of substance use (*F*(5.22, 350.68) = 0.49, *p* = .789), and craving (*F*(5.39, 139.90) = 0.34, *p* = .899). Fourth, we excluded participants from the analysis who reported no days of substance use in the past 30 days at baseline. Patients who already stopped consuming alcohol/cannabis before the start of their therapy can logically not further decrease their use. This could have biased the results, especially because there were double as many non-using participants in the ABM group (*n* = 10) compared with the control group (*n* = 5). The results showed that also when excluding these participants from the analysis, there was no significant interaction of time by condition for the frequency of used substance (*F*(2.70, 280.82) = 0.29, *p* = .810), or craving (*F*(2.71, 144.69) = 0.88, *p* = .444). Finally, we tested the effects of ABM intervention on the number of standard units of alcohol (only including AUD patients). There was no significant interaction between time and condition (*F*(2.05, 582.16) = 0.44, *p* = .649), again indicating a similar pattern between both groups.

After conducting the study, it turned out that the questions with regard to relapse lacked sufficient sensitivity, especially because of the diversity in treatment goal (i.e., moderation or abstinence). In addition, we had a high percentage of drop-out. Therefore, there was no solid base to conduct a Cox-regression analysis as was planned and described in the study protocol [37]. However, to more specifically investigate whether there were long term differences between the ABM group and the control group, we conducted additional RM-ANOVAs in which we only compared baseline values with 12 FU values. For the frequency of used substance, there was no significant interaction of time by condition in the overall sample (*F*(1, 448.54) = 0.44, *p* = .508). Also when adding the type of substance to the model, the three-way interaction remained non-significant (*F*(1, 435.98) = 0.40, *p* = .528). In line, for craving, there was no significant difference between the conditions when comparing baseline with 12 FU (*F*(1, 135.52) = 0.63, *p* = .430), and this result remained non-significant when type of substance was added to the model (*F*(1, 327.50) = 0.57, *p* = .451). Finally, when testing long term effects of ABM on the number of standard units of alcohol when only including participants with a diagnoses of AUD, no significant interaction between time and condition was found (*F*(1, 1362.92) = 0.19, *p* = .663).

## Discussion

Even after initial successful treatment, patients diagnosed with substance use disorders often relapse [49]. Given the need for more effective treatment in addiction care, the current study aimed to test whether (long-term) treatment outcome can be improved by adding an attentional bias modification (ABM) component to the intervention. A novel ABM intervention was provided as a home-delivered, and internet-based multi-session training to outpatients diagnosed with alcohol use disorder (AUD) or cannabis use disorder (CUD) as an add-on to treatment as usual (TAU; i.e., CBT-based intervention). In particular, we tested whether the ABM training could augment the effects of TAU by further reducing substance use and craving, and by reducing rates of relapse 6 and 12 months after the end of treatment. In contrast to the hypothesis that ABM would yield additional therapeutic benefit, there was no difference between patients who received the ABM intervention and those who received the control condition with regard to substance use (i.e., frequency and for AUD patients also quantity) and craving at post-test, or at 6 or 12 months follow-up. Additionally, the ABM intervention had no clinically relevant effects on substance use and craving when examining participants who completed a substantial number of ABM training sessions (a minimum of six sessions). Further, the effects of the ABM intervention on the frequency of substance use and on craving did not differ between the two types of substance use disorder. With regard to relapse, the addition of the ABM intervention to TAU had no effect on the duration until relapse took place. There were also no differences in percentage of patients who reported relapse(s) within 12 months post-treatment. On average, participants in all conditions showed reduced frequency of substance use and craving at post-test. Thereafter, the frequency of substance use showed no further significant changes, whereas for craving there was a significant increase from post-test to 6 months follow-up. Comparable with the results of craving, secondary physical and psychological complaints generally reduced from baseline to post-test, and increased again from post-test to 6 months follow-up.

### Effects of ABM intervention on treatment outcome

The current study found no support for the idea that the addition of ABM intervention to CBT-based TAU, would serve to improve treatment outcome for AUD or CUD outpatients in terms of reduced substance use and craving. One explanation for the non-significant findings is that clinical changes may depend on the successful modification of AB [50], and the BITT intervention may not have had a sufficient impact on patients’ AB. In line with this, there was no significant effect of ABM condition on AB as indexed by the OOOT. However, the high error rate of the OOOT may have prevented it from having sufficient sensitivity to adequately capture the effects of the BITT on AB [51]. Future studies may benefit from using an improved version of the OOOT (for recommendations, see [52]) or an alternative AB task with better psychometric properties (for example based on eye-tracking procedures such as described in [53]) to index the impact of ABM on individuals’ AB.

Another explanation for the non-significant findings on treatment outcome is that the BITT may not have targeted the attentional process(es) implicated in addiction symptomology. It seems reasonable to assume that the BITT approach to ABM may primarily targeting difficulty disengaging attention from substance-relevant cues. Participants need to consistently disengage their attention from substance-relevant cues in order to track the single substance-irrelevant cue. Support for this idea can be found in a previous study showing that completing a food version of the BITT resulted in a significant reduction of disengagement bias but not engagement bias [54]. It might thus be that disengagement bias is not causally related to addiction, and that therefore the current ABM intervention did not result in clinically relevant changes. It could then still be that modifying engagement bias instead of disengagement bias might be helpful to reduce substance use. However, this seems not very plausible as previous research using the same assessment task as in the present study showed that alcohol consumption was related to the strength of disengagement bias whereas the amount of consumption appeared unrelated to engagement bias [52].

The current study found no evidence that the BITT component served to reduce relapse. That is, patients who received the ABM intervention did not report lower rates of relapse than patients who did not receive the intervention, and there were no differences in the duration until relapse. Similarly, no long-term differences on substance use and craving were found between groups. These findings contrast with previous studies that have found effects of cognitive bias modification interventions on relapse [34–36, 55]. In addition to the explanations given above, this may reflect the discrepancy between the very different type of CBM procedure adopted in the present study compared to those used in these previous studies. Another explanation for the differences between the current findings and the findings of previous studies might regard the fact that previous studies have tested the effectiveness of the interventions in patients who were admitted and treated in a clinical setting, whereas the patients in the current study received outpatient treatment. The patients in the current sample might therefore differ to patients in previous studies, for example with regard to the severity of addiction and comorbidity. In addition, the differences in findings might be due to the fact that in contrast to previous studies, in the current study patients either intended moderation of alcohol/cannabis use or abstinence. As a result, no straightforward assessment of relapse was possible. Further, as the current study did not record the patients’ personal treatment goal, possible differences could not be tested. Therefore, we cannot rule out that the effectiveness of ABM intervention is dependent on the goal of treatment, especially because a recent meta-analysis found that cognitive bias modification might possibly only be effective when abstinence is the goal of treatment [29].

### Credibility of ABM training and participant motivation

After completing a practice session, the expectations with regard to both trainings, active and placebo, were comparable. In both groups around 60% of the patients expected the training to have a positive influence on their attention, to help them with moderation or abstinence, and to have a positive influence on their overall treatment outcome. Clearly, these results corroborate the credibility of the placebo condition. Besides that, it suggests that a slight majority of patients believed computerized interventions to be helpful in their treatment. However, it also points to the fact that around 40% did not believe in the added value of such an intervention. In line, after treatment, the extent to which the training was experienced as positive and the motivation to train on a regular basis appeared to be rather mixed. This might also explain why the compliance with the training in terms of completed training sessions varied across patients. However, lack of compliance and motivation is no problem of computerized interventions in particular, but a more common problem in therapy, possibly especially in substance use disorders (see for example [56, 57]). In future studies it seems highly relevant to further increase motivation of patients to comply to their treatment, for example by means of individualization to better satisfy the needs of the patients, especially as compliance has been found to be an important predictor of treatment success [58].

### Effects of treatment as usual on treatment outcome

The current findings emphasize the importance of improving treatment outcome in substance use disorders. Within one year after treatment, around 60–65% of patients in the current study reported to have experienced a relapse. This finding is in line with previous literature, suggesting that up to 50% of patients treated for substance use disorders relapse within the first year after treatment [49]. Further, the findings of the current study point to the idea that protocolized CBT is able to reduce relevant symptoms, but that these effects are on average not long lasting. That is, craving and secondary physical and psychological complaints increased from post-test to 6 months follow-up, with a tendency of being even higher than before the start of treatment. This increase of relevant symptoms, and craving in particular, might trigger relapse. It is important, therefore, to improve treatment outcome in a way that relevant symptoms remain low for a longer period of time and thereby reducing the relapse rates.

### Strengths and limitations

The current study has several strengths such as the inclusion of a clinical sample of treatment-seeking individuals, the addition of ABM intervention as an add-on to TAU, the accessibility by providing the ABM intervention in the home-environment, the involvement of the therapists to motivate the patients, and the long-term follow-up period until 12 months after end of treatment. There are also some limitations that may bear on the interpretation of the results. First, as described above, the results with regard to relapse might be influenced by the diversity in treatment goals (i.e., moderation or abstinence), and the related subjectivity with which participants might have answered the relapse-relevant questions. Thus, we cannot rule out the possibility that the findings might have looked different if relapse had been operationally defined with respect to patients’ own treatment goal. Future studies could either reduce variation due to differing patient goals by including only patients who intend to stay abstinent, or else could take account of such variation by collecting data on participants’ treatment goals, and computing treatment success and relapse with respect to these goals.

Second, participants diagnosed with AUD and CUD were combined for the analyses which could have influenced the results if the ABM intervention was effective for one disorder but not for the other. However, given that AB has been found to be associated with treatment outcome in both substance use disorders [59, 60], we assumed that the ABM intervention would have a similar effect for both substance use disorders. Indeed, the current results indicated that there was no difference between patients diagnosed with AUD and CUD with regard to the effects of the ABM intervention. Nevertheless, given possible power issues of the current study to find such a difference (rather small sample of patients with CUD), we cannot rule out that effects of ABM interventions differ between different substance use disorders, especially as most research has focused on AUD, and relatively little is known about the effects in other addictions.

Third, the current study aimed to deliver ABM as an integrated add-on to TAU by actively involving the therapist, but it may not have been the case that therapists integrated the ABM intervention sufficiently. The findings suggest that therapists greatly varied in their tendency to integrate the ABM intervention in the TAU sessions. Although we only had very limited data from the therapists themselves, the data indicated that therapists’ judgment on whether or not they found the ABM intervention to be effective varied a lot. This might explain the variation in compliance with the research protocol. Although the current study invested in the compliance of therapists in several ways (e.g., by providing them with relevant information about the rationale of the study and the study itself, both via a training and a written protocol), it might be important for future studies to further improve the motivation of therapists to adhere to the protocol, for example by organizing short booster meetings in which the rationale and relevance of the study is repeated.

Finally, there was great variability in the number of completed ABM training sessions in the current sample, varying from zero to 45 sessions. On average, patients completed around 12 sessions of ABM, which translates into approximately 120 minutes of training. With regard to the duration in minutes, this training intensity is comparable with a previous study that found effects of a similar intervention on relapse [35]. However, in the study of Rinck and colleagues [35] patients completed six sessions of cognitive bias modification training, and each session approximately lasted twice as long as the sessions of the current study (resulting in the same amount of time spent for the training). It might be that in order to effectively target processes such as AB, not only the number of completed training sessions is relevant, but also the intensity of each single session to allow appropriate consolidation. Future studies should further investigate which intensity of such interventions is necessary in order to achieve clinically relevant effects.

## Conclusion

Based on this RCT in the Dutch population, the current findings provided no support for the hypothesis that a multi-session ABM intervention as an add-on to CBT-based TAU can contribute to treatment outcome in outpatients diagnosed with alcohol or cannabis use disorders. This raises questions regarding the relevance of AB as a target for treatment in substance use disorders. It can, however, also be that ABM only has an effect when combined with an abstinence treatment goal (cf., [35]), or that AB is more persistent than expected, and therefore difficult to change with relatively short interventions such as the current ABM procedure. In addition, it could be that the current ABM did not target the most critical component of AB. Clearly, then, future studies are needed to more precisely delineate the role of engagement and disengagement bias in the persistence of addiction, and to improve insight in how these biases can be optimally targeted as a means to improve long-term treatment outcome in substance use disorders. Future studies should further invest in the inclusion of higher sample sizes, and/or combining other populations, for example from other countries, to determine the effectiveness of ABM intervention.

## Supporting information

S1 Appendix Imputation script and example script of RM-ANOVA in SPPS and pooling in R. (DOCX)

S2 Appendix Link to imputation model in excel file. (DOCX)

S3 Appendix Table of means and standard deviations of the relevant group descriptives, attentional bias indices and primary outcome variables. (DOCX)

S1 Protocol (PDF)

S1 Checklist CONSORT 2010 checklist of information to include when reporting a randomised trial*. (DOC)
