# Supplementary material for: Effectiveness of attentional bias modification training as add-on to regular treatment in alcohol and cannabis use disorder: A multicenter randomized control trial
Source: PLoS One. 2021 Jun 4;16(6):e0252494. doi: 10.1371/journal.pone.0252494 (PMC8177423; doi:10.1371/journal.pone.0252494)
Supplement: S1 Appendix — (DOCX) [file pone.0252494.s001.docx]

**S1 Appendix. Imputation script and example script of RM-ANOVA in SPPS and pooling in R.**

**Imputation script:**

#----------------------------------------------------------------------------------

# Impute with mice: all data

# Impute with defaults

#----------------------------------------------------------------------------------

ini <- mice(data, maxit = 0, print = F)

# Specify imputation methods

meth <- ini$method

# Passive imputation for variables dag_b to dag_12f; by default not imputed but computed

meth["dag_b"] <- "~I(al_dag_b + ca_dag_b)"

meth["dag_p"] <- "~I(al_dag_p + ca_dag_p)"

meth["dag_6f"] <- "~I(al_dag_6f + ca_dag_6f)"

meth["dag_12f"] <- "~I(al_dag_12f + ca_dag_12f)"

# Passive imputation for variables ooot_eng_b to ooot_dis_12f

meth["ooot_eng_b"] <- "~I(ooot_neut_b - ooot_targ_b)"

meth["ooot_dis_b"] <- "~I(ooot_distr_b - ooot_neut_b)"

meth["ooot_eng_p"] <- "~I(ooot_neut_p - ooot_targ_p)"

meth["ooot_dis_p"] <- "~I(ooot_distr_p - ooot_neut_p)"

meth["ooot_eng_6f"] <- "~I(ooot_neut_6f - ooot_targ_6f)"

meth["ooot_dis_6f"] <- "~I(ooot_distr_6f - ooot_neut_6f)"

meth["ooot_eng_12f"] <- "~I(ooot_neut_12f - ooot_targ_12f)"

meth["ooot_dis_12f"] <- "~I(ooot_distr_12f - ooot_neut_12f)"

# Change method to linear regresion, based on normality for few variables:

meth[c("verl_b", "verl_p", "verl_6f", "verl_12f")] <- "norm"

meth[c("ooot_distr_b", "ooot_targ_b", "ooot_neut_b")] <- "norm"

meth[c("ooot_distr_p", "ooot_targ_p", "ooot_neut_p")] <- "norm"

meth[c("ooot_distr_6f", "ooot_targ_6f", "ooot_neut_6f")] <- "norm"

meth[c("ooot_distr_12f", "ooot_targ_12f", "ooot_neut_12f")] <- "norm"

# Leave rest default: ppm

meth

# Specify predictors in imputation model

pred <- ini$predictorMatrix

write.table(pred, "pred.dat") # write to file and adapt in Excel

# Specify predictor matrix imputation models - in Excel and imported back in R

# Variables not used to impute: ppn, cond2

# All variables that are not imputed: ppn, cond, cond2, mid, tsess, lft, gesl, opl, beh, beh5j, fam1, fam2

# Computed variables are not used to impute: dag_b, dag_p, dag_6f, dag_12f

# One exception: dag_b, dag_p, dag_6f, dag_12f are used to impute terugv - warning message in logged events when imputing terugv with original variables

# Computed variables are not used to impute: ooot_eng_b, ooot_dis_b, ooot_eng_p, ooot_dis_p, ooot_eng_6f, ooot_dis_6f, ooot_eng_12f, ooot_dis_12f

# No cross-lag interactions between variables

pred_new <- as.matrix(read.table(file = "pred_new.csv", header = T, sep=";", row.names = 1))

# Check some of the variables

# pred_new["dass_b",]

# pred_new["terugv",]

# pred_new["al_dag_b",]

#----------------------------------------------------------------------------------

# Impute

#----------------------------------------------------------------------------------

m <- 50

mit <- 20

dat_mi <- mice(data, m = m, method = meth, pred = pred_new, maxit = mit, seed = 1212665)

dat_mi$loggedEvents

**Example script of RM-ANOVA in SPPS and pooling in R:**

# RM-ANOVA in SPSS

GLM <within-subject variables> BY <factor>

/WSFACTOR=Time 4 REPEATED

/METHOD=SSTYPE(3)

/EMMEANS=TABLES(Time) COMPARE ADJ(BONFERRONI)

/PRINT=DESCRIPTIVE HOMOGENEITY

/CRITERIA=ALPHA(.05)

/WSDESIGN=Time

/DESIGN= <factor>.

#----------------------------------------------------------------------------------

# Pooling SPSS analyses results in R

# First saving SPSS tables as data sets, then reading these data sets in R

#----------------------------------------------------------------------------------

# Function for pooling F values

pool.F <- function(F, df1, m=length(F)){

d <- df1*F

r2 <- (1 + 1/m) * var(sqrt(d))

D2 <- ((mean(d)/df1) - ((m+1)/(m-1)) * r2) / (1 + r2)

df2 <- df1^(-3/m) * (m-1) * (1 + (1/r2))^2

p <- 1 - pf(D2, df1, df2, lower.tail = T)

result <- c(D2, df1, df2, p)

names(result) <- c("F","df1","df2","p")

return(result)

}

# Function for pooling F values

pool.F.adj <- function(F, df1, m=length(F)){

d <- df1*F

r2 <- (1 + 1/m) * var(sqrt(d))

D2 <- ((mean(d)/df1) - ((m+1)/(m-1)) * r2) / (1 + r2)

df2 <- df1^(-3/m) * (m-1) * (1 + (1/r2))^2

#----- adjust df2 (Reiter, 2007) -----

k <- df1

v_c <- df2

v_cs <- ((v_c + 1)/(v_c + 3))*v_c

t <- k*(m - 1)

a <- r2 * t/(t - 2)

t4 <- 1/(t - 4)

v2 <- v_cs - 2*(1 + a)

v4 <- v_cs - 4*(1 + a)

z <- 1/v4 + t4*((a^2*v2) / ((1 + a)^2*v4)) + t4*( ((8*a^2*v2)/((1 + a)*v4^2)) + ((4*a^2)/((1 + a)*v4)) ) +

t4*( ((4*a^2)/(v4*v2)) + ((16*a^2*v2)/(v4^3)) ) + t4*( (8*a^2)/(v4^2) )

v_f <- 4 + 1/z

#-------------------------------------

p <- 1 - pf(D2, k, v_f, lower.tail = T)

result <- c(D2, k, v_f, p)

names(result) <- c("F","df1","df2","p")

return(result)

}
