# Supplementary material for: Effectiveness of attentional bias modification training as add-on to regular treatment in alcohol and cannabis use disorder: A multicenter randomized control trial
Source: PLoS One. 2021 Jun 4;16(6):e0252494. doi: 10.1371/journal.pone.0252494 (PMC8177423; doi:10.1371/journal.pone.0252494)
Supplement: S2 Appendix — (DOCX) [file pone.0252494.s002.docx]

**S2 Appendix. Link to imputation model in Excel file.**

Link to Excel file in which imputation model is specified:

https://dataverse.nl/dataset.xhtml?persistentId=hdl:10411/UICVVD
