# Supplementary material for: Effectiveness of attentional bias modification training as add-on to regular treatment in alcohol and cannabis use disorder: A multicenter randomized control trial
Source: PLoS One. 2021 Jun 4;16(6):e0252494. doi: 10.1371/journal.pone.0252494 (PMC8177423; doi:10.1371/journal.pone.0252494)
Supplement: S3 Appendix — (DOCX) [file pone.0252494.s003.docx]

**S3 Appendix**

Table

*Means and standard deviations of the relevant group descriptives, attentional bias indices and primary outcome variables.*

|  | | ABM group (*n* = 77) | | | | Placebo subgroup (*n* = 33) | | | | TAU-only subgroup (*n* = 32) | | | |
| --- | --- | --- | --- | --- | --- | --- | --- | --- | --- | --- | --- | --- | --- |
| Age | | 44.51 (13.37) | | | | 45.36 (13.82) | | | | 43.78 (15.49) | | | |
| Gender | | 71.4 % male | | | | 78.8 % male | | | | 68.8 % male | | | |
| Alcohol use disorder | 77.9 % | | | | | 81.8 % | | | | 75.0 % | | | |
| Cannabis use disorder | | 22.1% | | | | 18.2 % | | | | 25.0 % | | | |
|  | | *Baseline* | *Post-test* | *6 FU* | *12 FU* | *Baseline* | *Post-test* | *6 FU* | *12 FU* | *Baseline* | *Post-test* | *6 FU* | *12 FU* |
| Engagement index | | -107  (641) | -367  (698) | -37  (1223) | -184  (794) | -239 (810) | -355 (719) | -336 (1410) | -295 (860) | -75 (761) | -280 648) | -537 (1087) | -244 (778) |
| Disengagement index | | 783  (970) | 1071  (1013) | 1045  (1257) | 927 (1132) | 420 (1082) | 703 (1035) | 845 (1385) | 909 (1292) | 748 (971) | 701 (837) | 855 (1250) | 972 (1169) |
| Frequency substance use | | 17.92 (11.49) | 7.72 (11.03) | 8.82 (10.58) | 8.97 (10.66) | 17.35 (12.40) | 7.41 (8.96) | 10.14 (11.19) | 11.48 (11.42) | 23.03 (10.02) | 10.85 (11.40) | 13.31 (12.12) | 13.93 (12.76) |
| Craving | | 7.23 (3.44) | 4.34 (4.40) | 9.42 (4.03) | 9.43 (4.51) | 6.55 (4.58) | 5.35 (4.80) | 9.63 (4.34) | 9.73 (4.90) | 7.66 (3.76) | 5.84 (4.94) | 9.82 (4.54) | 10.14 (4.10) |
