# Supplementary material for: Effectiveness of attentional bias modification training as add-on to regular treatment in alcohol and cannabis use disorder: A multicenter randomized control trial
Source: PLoS One. 2021 Jun 4;16(6):e0252494. doi: 10.1371/journal.pone.0252494 (PMC8177423; doi:10.1371/journal.pone.0252494)
Supplement: S1 Protocol — (PDF) [file pone.0252494.s004.pdf]

RESEARCH PROTOCOL

PROTOCOL TITLE 'E-health attentional bias modification training as add-on to regular treatment in alcohol or cannabis dependent outpatients'

**Protocol ID**	**NL54435.042.15**
**Short title**	**E-health attentional bias intervention in addiction**
**EudraCT number**	***<only applicable for studies with an investigational medicinal product>***
**Version**	**2**
**Date**	**18-03-2016**
**Coordinating investigator/project leader**	***Dr. Madelon van Hemel-Ruiter***<br>***Senior Researcher***<br>***Verslavingszorg Noord Nederland***<br>***Postbus 8003, 9702 KA Groningen***
**Principal investigator(s) (in Dutch: hoofdonderzoeker/ uitvoerder)**<br>***<Multicenter research: per site>***	***Janika Heitmann, MSc.***<br>***PhD Student***<br>***Verslavingszorg Noord Nederland***<br>***Postbus 8003, 9702 KA Groningen***<br>***050-3648900***<br><br>***Martine Fledderus,***<br>***Researcher Tactus Verslavingszorg***<br>***Postbus 154, 7400 AD Deventer***<br>***088 382 28 87***<br><br>***Laura deFuentes-Merillas***<br>***Senior Wetenschappelijk Medewerker***<br>***Novadic-Kentron, Research & Development***<br>***Hogedwarsstraat 3, 5261 LX Vught***

	073-684 9500
**Sponsor (in Dutch: verrichter/opdrachtgever)**	***VNN***
**Subsidising party**	***ZonMw***
**Independent expert (s)**	***Dr. Eric Blaauw***<br>***Senior Onderzoeker***<br>***Verslavingszorg Noord Nederland***<br>***Postbus 8003, 9702 KA Groningen***<br>***Tel 050 3648900***
**Laboratory sites <*if applicable*>**	***Not applicable***
**Pharmacy <*if applicable*>**	***Not applicable***

PROTOCOL SIGNATURE SHEET

Name	Signature	Date
<For non-commercial research,><br>**Head of Department:**<br>***<include name and function>***	**Dr. Ingrid Willems**<br>**Manager Zorg, VNN**	**18-03-2016**
**[Coordinating Investigator/Project leader/Principal Investigator]:**<br>***<please include name and function>***	**Dr. Madelon van Hemel**<br>**Senior onderzoeker VNN**	**18-03-2016**

TABLE OF CONTENTS

1. INTRODUCTION AND RATIONALE 11
2. OBJECTIVES 13
3. STUDY DESIGN 13
4. STUDY POPULATION 16
4.1 Population (base) 16
4.2 Inclusion criteria 16
4.3 Exclusion criteria 16
4.4 Sample size calculation 16
5. TREATMENT OF SUBJECTS 17
5.1 Investigational product/treatment 17
5.2 Use of co-intervention (if applicable) 17
5.3 Escape medication (if applicable) 20
6. INVESTIGATIONAL PRODUCT 20
6.1 Name and description of investigational product(s) 20
6.2 Summary of findings from non-clinical studies 20
6.3 Summary of findings from clinical studies 20
6.4 Summary of known and potential risks and benefits 20
6.5 Description and justification of route of administration and dosage 20
6.6 Dosages, dosage modifications and method of administration 20
6.7 Preparation and labelling of Investigational Medicinal Product 20
6.8 Drug accountability 20
7. NON-INVESTIGATIONAL PRODUCT 21
7.1 Name and description of non-investigational product(s) 21
7.2 Summary of findings from non-clinical studies 21
7.3 Summary of findings from clinical studies 21
7.4 Summary of known and potential risks and benefits 21
7.5 Description and justification of route of administration and dosage 21
7.6 Dosages, dosage modifications and method of administration 21
7.7 Preparation and labelling of Non Investigational Medicinal Product 21
7.8 Drug accountability 21
8. METHODS 22
8.1 Study parameters/endpoints 22
8.1.1 Main study parameter/endpoint 22
8.1.2 Secondary study parameters/endpoints (if applicable) 22
8.1.3 Other study parameters (if applicable) 22
8.2 Randomisation, blinding and treatment allocation 22
8.3 Study procedures 23
8.4 Withdrawal of individual subjects 25
8.4.1 Specific criteria for withdrawal (if applicable) 25
8.5 Replacement of individual subjects after withdrawal 25
8.6 Follow-up of subjects withdrawn from treatment 25

8.7 Premature termination of the study....25
9. SAFETY REPORTING....26
9.1 Section 10 WMO event....26
9.2 AEs, SAEs and SUSARs....26
9.2.1 Adverse events (AEs)....26
9.2.2 Serious adverse events (SAEs)....26
9.2.3 Suspected unexpected serious adverse reactions (SUSARs)....27
9.3 Annual safety report....27
9.4 Follow-up of adverse events....27
9.5 [Data Safety Monitoring Board (DSMB) / Safety Committee]....27
10. STATISTICAL ANALYSIS....28
10.1 Primary study parameter(s)....28
10.2 Secondary study parameter(s)....28
10.3 Other study parameters....29
10.4 Interim analysis (if applicable)....29
11. ETHICAL CONSIDERATIONS....30
11.1 Regulation statement....30
11.2 Recruitment and consent....30
11.3 Objection by minors or incapacitated subjects (if applicable)....31
11.4 Benefits and risks assessment, group relatedness....31
11.5 Compensation for injury....31
11.6 Incentives (if applicable)....32
12. ADMINISTRATIVE ASPECTS, MONITORING AND PUBLICATION....33
12.1 Handling and storage of data and documents....33
12.2 Monitoring and Quality Assurance....33
12.3 Amendments....33
12.4 Annual progress report....34
12.5 End of study report....34
12.6 Public disclosure and publication policy....34
13. STRUCTURED RISK ANALYSIS....36
13.1 Potential issues of concern....36
13.2 Synthesis....36
14. REFERENCES....36

LIST OF ABBREVIATIONS AND RELEVANT DEFINITIONS

**AB**	**Attentional bias**
**ABR**	**ABR form, General Assessment and Registration form, is the application form that is required for submission to the accredited Ethics Committee (In Dutch, ABR = Algemene Beoordeling en Registratie)**
**AE**	**Adverse Event**
**AR**	**Adverse Reaction**
**AUD**	**Alcohol Use Disorder**
**CA**	**Competent Authority**
**CCMO**	**Central Committee on Research Involving Human Subjects; in Dutch: Centrale Commissie Mensgebonden Onderzoek**
**CUD**	**Cannabis Use Disorder**
**CV**	**Curriculum Vitae**
**DSMB**	**Data Safety Monitoring Board**
**EU**	**European Union**
**EudraCT**	**European drug regulatory affairs Clinical Trials**
**GB-GGZ**	**Basis GGZ**
**GCP**	**Good Clinical Practice**
**iABM**	**Internet-based attentional bias modification**
**IB**	**Investigator's Brochure**
**IC**	**Informed Consent**
**IMP**	**Investigational Medicinal Product**
**IMPD**	**Investigational Medicinal Product Dossier**
**METC**	**Medical research ethics committee (MREC); in Dutch: medisch ethische toetsing commissie (METC)**
**(S)AE**	**(Serious) Adverse Event**
**SPC**	**Summary of Product Characteristics (in Dutch: officiële productinfomatie IB1-tekst)**
**Sponsor**	**The sponsor is the party that commissions the organisation or performance of the research, for example a pharmaceutical company, academic hospital, scientific organisation or investigator. A party that provides funding for a study but does not commission it is not regarded as the sponsor, but referred to as a subsidising party.**
**SUSAR**	**Suspected Unexpected Serious Adverse Reaction**
**TAU**	**Treatment as Usual**

**Wbp**	**Personal Data Protection Act (in Dutch: Wet Bescherming Persoonsgevens)**
**WMO**	**Medical Research Involving Human Subjects Act (in Dutch: Wet Medisch-wetenschappelijk Onderzoek met Mensen**

SUMMARY

Rationale: Addiction is characterized by high relapse numbers after successful treatment. Cognitive models of addiction suppose that the development and maintenance of addiction can be explained by an imbalance between automatic, impulsive processes and reflective, controlling processes. Research has shown that addicted individuals demonstrate a heightened automatic attention (attentional bias, AB) for ‘their’ substance. That is, their attention is automatically and unintentionally drawn towards information that is related to their addiction, and they have trouble redirecting their attention away from this information. Using computer tasks the strength of this AB can be measured. stronger AB has been associated with greater severity of addiction, poorer treatment outcomes, and increased relapse. Recently, new techniques have been developed in order to train the automatic attention away from substance information (attentional bias modification, ABM). Importantly, ABM can be effectively delivered via the Internet (iABM), which allows a low-cost, efficient delivery system for this treatment. The proposed project will investigate the (cost)effectiveness of iABM as an add-on to addiction treatment as usual (TAU) (i.e., blended therapy).

We hypothesize that patients receiving iABM as add-on to TAU, will show less addiction problems, decreased relapse, increased health, reduced physical and psychological complaints, and reduced use of health care post-intervention and at 6 & 12 month FU. Further, we hypothesize that the effects on the individual and societal level cause a decrease in societal costs that outweighs the additional costs of the iABM.

Objective:

The proposed project will examine the potential benefit of adding iABM to TAU for AUD and CUD patients in GB-GGZ addiction care on the health outcomes related to the increase in costs for adding this intervention to treatment as usual. Therefore, the project was designed to examine the following questions:

1- Does adding iABM to TAU for AUD and CUD patients at GB-GGZ lead to a more sustained decrease in addiction problems as reflected by lower post-intervention, and 6 & 12 month level of substance use, dependency, craving and relapse rates?

2- Does adding iABM to TAU for AUD and CUD patients at GB-GGZ enhance their broader health, and reduce their use of other health care resources at 6 & 12 month follow-up?

3- Do the economic benefits of adding iABM to TAU at GB-GGZ outweigh the costs?

A secondary aim of this project is to explore which type of patients benefit most from the addition of internet-based ABM, in order to enable the most effective targeted delivery of this treatment component in the future.

Study design:

In an RCT study with pre- post- design and 6 & 12-month FU, patients will be assigned to TAU+iABM condition, or control condition (half TAU+placebo iABM, half TAU only).

Study population:

Adult patients diagnosed with AUD or CUD admitted to GB-GGZ of VNN, Tactus or Novadic-Kentron

Intervention (if applicable):

The iABM is a computerized intervention aimed at modifying automatically triggered attentional processes that have been shown to play a role in addiction During this intervention patients will be shown moving images of alcohol (cannabis) on a computerscreen, together with neutral moving images. They are instructed to follow the neutral images with the cursor of the mouse, and thus, to neglect the alcohol (cannabis) images. The iABM will be delivered at home, in multiple sessions concurrent with and for the duration of TAU.

Main study parameters/endpoints:

Primary outcome parameters are changes in substance use, level of dependency and craving, and relapse-rates. Health condition, and societal costs will be primary outcome parameters to assess cost-effectiveness of this intervention.

Nature and extent of the burden and risks associated with participation, benefit and group relatedness:

We expect a direct and longer-term benefit for the iABM group on top of effects of TAU (and to a lesser extent for the placebo training group), related to a small investment (short sessions, at home environment), and a very limited (possibly even absent) risk (no extra travels, no medication, possible (short) increase of craving by demonstration of pictures of alcohol and cannabis, but certainly not stronger triggers than the 'daily life' triggers. Further, we expect no extra benefit on top of TAU for the control group, related to a minimum investment of four times a 20-30 minute assessment, and no potential risk factors.

1. INTRODUCTION AND RATIONALE

With a life-time prevalence of 19%, alcohol and drug addiction is the most common psychiatric disease after anxiety and depression. In 2013, 66.000 people in the Netherlands were treated for addiction, and for half of them this was not their first time in treatment. This underlines the persistent character of addiction, and the need for (cost)effective interventions that deliver good outcomes with low relapse rates. This need is recently underscored by the reports of RIVM (1) and ZiN (2), calling for more research investigating the (cost) effectiveness of addiction treatments.

One possible reason of the limited success rates of current interventions might be their focus on conscious appraisals and teaching people to improve rational decision making. Current dual process models of addiction (e.g., 3), emphasize that next to these more explicit, deliberate processes, also more automatic, implicit processes are critically involved in the persistence of addictive behaviors and in triggering relapse. Several studies support the view that an automatically triggered attentional bias (AB) to substance-related cues may underlie compulsive drug use. First, there is evidence for a reciprocal relationship between AB and craving (4,5), and for a predictive role of AB for subsequent substance use (6). Further, stronger AB has been associated with greater severity of addiction (7,8), poorer treatment outcomes (9), and increased relapse (10,11,12).

Laboratory studies have shown that AB can be effectively altered by computerized Attention Bias Modification (ABM) procedures (13). Recently, these types of procedures have been evaluated as clinical tools, intended to attenuate dysfunctional clinical symptoms by reducing AB. The initial ABM studies predominantly focused on anxiety disorders, and confirmed that ABM procedures designed to reduce AB to threat cues served to decrease anxiety symptoms. More recently, the ABM approach has also been successfully applied to reduce attention to substance-related cues in addicted people. That is, there is preliminary evidence that substance dependent individuals can benefit from ABM interventions when presented alone (14) or combined with Cognitive Behavioral Therapy (CBT;15). Therefore, it seems likely that the efficacy of traditional CBT can be increased by adding treatment components designed to attenuate the attentional capture of substance-related cues.

Importantly, ABM can be effectively delivered via the Internet (iABM), which allows a low-cost, efficient delivery system for this treatment. Moreover, this enables patients to train their AB in their own environment at precisely these moments when the patient experiences craving, and the urge to use alcohol or cannabis is high. Since cue-elicited craving is highly context-dependent, the delivery of iABM in the home-environment may well contribute to its efficacy. The proposed project will investigate the (cost)effectiveness of iABM as an add-on to addiction treatment as usual (TAU) (i.e., blended therapy). As such the proposed project perfectly fits with the recent call from the RIVM for research evaluating the cost-effectiveness of e-health interventions in the field of addiction.

Recently, promising results were found testing ABM in subclinical and a clinical group of substance abusers, showing a decrease in AB, craving and/or use (16, 17, 18, 19). Furhter, a recent meta-analysis concluded that ABM seems promising but that the research in this field would benefit from more research in addicted populations with more challenging tasks in

settings in which use normally occurs, and when craving is elevated (20). The present proposal thus describes a study protocol designed to retrain AB in this kind of setting.

2. OBJECTIVES

The proposed project will examine the potential benefit of adding iABM to TAU for AUD and CUD patients in GB-GGZ addiction care on the health outcomes related to the increase in costs for adding this intervention to treatment as usual. Therefore, the project was designed to examine the following questions:

Primary objectives:

1- Does adding iABM to TAU for AUD and CUD patients at GB-GGZ lead to a more sustained decrease in addiction problems as reflected by lower post-intervention, and 6 & 12 month level of substance use, dependency, craving and relapse rates?
2- Does adding iABM to TAU for AUD and CUD patients at GB-GGZ enhance their broader health, and reduce their use of other health care resources at 6 & 12 month follow-up?
3- Do the economic benefits of adding iABM to TAU at GB-GGZ outweigh the costs?

Secondary objectives:
A secondary aim of this project is to explore which type of patients benefit most from the addition of internet-based ABM, in order to enable the most effective targeted delivery of this treatment component in the future.

3. STUDY DESIGN

In a randomized controlled trial (RCT), with a pre-post design, 213 treatment seeking adult patients diagnosed with alcohol use disorder (AUD) or cannabis use disorder (CUD) will be randomly assigned to either an iABM intervention + TAU (n = 107), or placebo training + TAU (n = 53) or TAU only (n = 53). The inclusion of two control groups enables the investigation of the effect of iABM + TAU related to TAU only, but also control for the placebo effect of adding an ehealth intervention as such to TAU. Participants will be recruited among patients who have been admitted to outpatient treatment of medium addiction problems (GB-GGZ) of three addiction treatment centers, i.e., VNN, Tactus or Novadic-Kentron.

TAU in the basis-GGZ consists of approximately 8-10 sessions of protocolled CBT, and when indicated, combined with a maximum of approximately 30% additional therapy time (e.g., medicine, psychotherapy). Together, patient and therapist will have one therapy session per week or per two weeks for a total period of 3 to 4 months. Total duration of therapy differs between patients, because it depends on the individual needs of the patient .

Patients will be included in the study before or just after their first appointment of regular treatment. The latest moment of inclusion is the second appointment of regular treatment. To promote participation, patients randomized into one of the control groups will be offered iABM after the end of FU2, so they will also profit from the benefits if proven effective. Standard treatment in the GB-GGZ consists of cognitive behavioral therapy (CBT) with the possibility of some additional individually adjusted treatment components (i.e., medication, or elements of anxiety/depression treatment). All participating patients will complete a pre-test, a post-test at the end of their regular treatment, and a 6-month and 12-month follow-up assessment.

After providing informed consent, the pre-test will be scheduled in their first or second week of treatment. Participating patients are asked to perform an attentional bias task at the computer (10 minutes), and to fill in the EQ-5D Health Questionnaire (16), and the TiC-P for costs associated with psychiatric illness (17) (10 minutes total). Components of the MATE (18) (Measurements in Addiction for Triage and Evaluation - questionnaire) will be assessed by the therapist as a part of the standard intake procedure at GB-GGZ. The components that will be used for the current project are: 1) Substance use, 4) Dependency and abuse, Q1) Craving (OCDS5), and Q2) Depression, Anxiety and Stress (DAS).

Patients who are assigned to the TAU condition receive their TAU as usual. Patients who are assigned to the iABM + TAU condition and patients assigned to the placebo-training + TAU receive their TAU as usual with the additional intervention. In the three weeks following the pretest, patients in the iABM or the placebo-training condition will complete a training session at home via the internet every day, during sessions of 10 minutes each. Across the next 3 weeks, sessions will be completed 3 times a week, and thereafter it will be completed once a week, until TAU comes to an end (mostly 3 to 4 months). In this way the training is adjusted

to TAU, following a staged pattern. Before and after each training session participants will score their craving on a VAS (visual analogue scale). Further, compliance will be automatically monitored, by following if, when and in which time-frame participants completed each training session.

At the end of the intervention, which coincides with the end of TAU, patients complete the post-test, including the same measures as the pre-test. The MATE will be assessed as a standard routine outcome measurement (ROM) by the therapist post-treatment (which coincides with the post-intervention moment). Further, therapists will be asked to fill-in a short evaluation form regarding their time investment in this study and possible direct positive or negative side effects they experienced from the training (5-10min).

Finally, 6 and 12 months after ending the intervention, patients will receive a follow-up assessment in the form of a computerized assessment, including an attentional bias task, MATE (parts 1,4, Q1 and Q2), Tic-P and EQ5D .

4. STUDY POPULATION

4.1 Population (base)

The study population consists of three patient groups, i.e., one receiving the iABM on top of their TAU (n = 107), one receiving TAU + placebo training (n=53) one receiving TAU only (n = 53). We will recruit 213 adults (18+ year) diagnosed with an alcohol use disorder (AUD) or a cannabis use disorder (CAD) who enter treatment at GB-GGZ of Tactus, Novadic- Kentron, or VNN. Patients who enter GB-GGZ treatment are diagnosed with a substance use disorder together with no or just mild single co-occurring psychiatric problems. These patients are not or minimally disabled at other areas of functioning. That is, they participate in a household, do have a job, and a social life

4.2 Inclusion criteria

In order to be eligible to participate in this study, a subject must meet all of the following criteria: individuals diagnosed with alcohol use disorder (AUD) and/or cannabis use disorder (CUD), who start GB-GGZ treatment in one of the three participating addiction treatment centres.

4.3 Exclusion criteria

Patients showing compulsive gaming, a gambling disorder, or internet addiction will be excluded from participation because the internet delivered training task might have counterproductive effects for this group of patients. Further, patients who do not have a Personal Computer (PC), or laptop and access to internet at home will be excluded for this study. Further, they will be excluded when not able to read and write fluently in Dutch.

4.4 Sample size calculation

We used G*power to calculate the needed sample size. To find a difference between groups of medium effect size with a power of 80% (alpha = 0.05), a sample of N = 64 per group (iABM training versus control (= alternative training or no training) suffices. The yearly influx is approximately 1200 patients per treatment center, with drop-out rates of approximately 20%. This would indicate that complete assessment of baseline and 1-yr follow-up can be finished within 2 years, even if drop-out rate is as high as 40% (review of home delivered ABM studies showed a mean drop-out rate of 23% after 4 months). In that worst-case scenario, an influx of 213 patients is needed.

5. TREATMENT OF SUBJECTS

The intervention that will be tested in this project is a computerized iABM training that will be added to TAU, aimed at modifying automatically triggered attentional processes that have been shown to play a role in substance dependency. The iABM will be delivered at home in multiple sessions, concurrently with and for the duration of TAU.In order to test whether it is likely that this iABM training would sort a decreasing effect on attentional bias, we started a pilot study among alcohol dependent outpatients of VNN. We used the findings of this study to improve the methodology of the current project (see also the attached documents K6 protocol pilot study and K6 results pilot study).

5.1 Investigational product/treatment

iABM training

The iABM is based on a recently developed dynamic ABM task (Bouncing Image Training Task-BITT; 21). In this task a series of 8 squares bounce around a computer screen. Within these squares, pictures of the addictive substance (i.e., alcohol or cannabis) are displayed, with the exception of one square that instead contains a benign picture unrelated to the addiction (e.g., a glass of soda). Participants completing the task must focus attention on the bouncing square that contains the benign picture, and track it with the cursor using the mouse (with good tracking leading to high “game scores” to sustain participants’ motivation). This task is complicated by the fact that the images in all squares change at frequent unpredictable time intervals. Most often the benign picture in the square being tracked will change into another benign picture (e.g., from water into 7-up into orange juice), meaning that the participant should continue to track this same bouncing square. However, at random times this benign image may instead change into a picture of the addictive substance, at which point a benign image appears in one of the other 7 squares not presently being tracked. When this happens, the participants should shift attention to the bouncing square where the benign image is now shown, and track this with the cursor using the mouse. With extended practice on this iABM task, people will systematically be trained to (1) keep attention focused on addiction-unrelated information, in a rapidly changing and complex environment that contains multiple addiction–relevant distracters and (2) to disengage attention from the presently attended locus as soon as addiction-related cues here.

Participants gain points which will be calculated per block. The scoring indicates the percentage of the total block-time one has followed the correct image in the current block. So the longer a person tracks the soda ball, the higher his score will be.

The game is divided in 4 blocks of 2.5 minutes (in the pilot study this training was over 15 minutes, which was found to be too long by almost half of the participants) . The substance of the target ball switches randomly within a programmed time-window. Scores are displayed after each block. In order to make it easier for participants to train the full 10 minutes, we built in some extra game-like elements, like high scores and more levels (also after we got to know in the pilot that many participants did find the length too long). During each block, the time is counting down in the upper right of the screen, and there is a bar on the top, showing an increasing green bar displaying the score. This game-like element of this intervention will motivate patients to do their best, and increase their scores with each block. When participants have followed the right block for 80% of the total time, a next level will be unlocked. The intervention will thus be individually adjusted based on performance, which makes it a challenging training throughout the full training period.

Placebo training

The placebo training is based on the same dynamic task, with this difference that half of the squares contain substance (i.e., alcohol or cannabis) pictures and half of the squares contain benign pictures. Every once in while one of the pictures (with evenly frequency a substance or a benign picture) will become lighter, and the participant then has to click on that particular picture with the cursor of the mouse. In this way attention will be trained, but no particular substance-related attention. In this way we will be able to control for a training effect.

Participants gain points which will be calculated per block. The scoring is based on the time participants need to ‘find’ the right block. So the shorter a person takes to find the correct block, the higher his score will be.

The game is divided in 4 blocks of 2.5 minutes. The substance of the balls switch randomly within a programmed time-window, with always having four substance and four benign balls. Scores are displayed after each block. During each block, the time is counting down in the upper right of the screen, and there is a bar on the top, showing an increasing green bar displaying the score. This game-like element of this intervention will motivate patients to do their best, and increase their scores with each block. When participants were faster than 20% of the total time to find the right block, a next level will be unlocked. The intervention will thus be individually adjusted based on performance, which makes it a challenging training throughout the full training period.

Both trainings

The training (iABM and placebo) will be delivered concurrently with TAU, for as long as TAU is continued. Across the first three weeks, sessions will be completed at home on a daily basis, with each session lasting 10 minutes. Across the next 3 weeks, sessions will be completed 3 times a week, and thereafter it will be completed once a week, until TAU comes to an end. In this way the training is adjusted to TAU, following a staged pattern. In consultation with the therapist, the patient will schedule the training sessions at the particular time of day when, for them, craving tend to be highest, thereby ensuring that training is delivered when the need is the highest, and benefits may be greatest. That is, preliminary research points to the idea that iABM will be only able to change behavior if this behavior is activated at the moment of training (22). As a welcome side-effect of this is, that patients are provided with an easy and quick tool to tackle their craving for a beer or joint at that specific difficult moment.

At the end of the pre-assessment, patients in the iABM or placebo intervention condition will be invited by email to start the online training, which is preceded by an online video instruction, and practice . This way patients are made familiar with the training. The system is developed in such a way, that patients only have to open a link that was send to them by email, by which the training will start. Scores, and levels of previous sessions are saved by the system and used to determine the level of the following session. This information is just send within the link, together with the patients unique participant number. Thus, patients do not have to change settings or fill-in their participant number. Further, they are provided with a help-desk telephone number and email address to ask for help when they are experiencing trouble with performing the task at home. To optimize compliance, patients will be sent an email and a text message to remind them to the session on each day they are due to complete ABM training. If needed, a reminder message will be sent. The system for automatically sending these messages is secured and for the project we will use participant numbers instead of patient numbers to guarantee privacy and security.

The therapist will assist the patient in identifying the best daily time point for the training session, and will actively encourage completion of training to maintain patient motivation. Therapist and patient will have weekly appointments, which will be (also) used to shortly evaluate the course of the sessions.

5.2 Use of co-intervention (if applicable)

The project is developed for patients in Generalistische Basis GGZ (GB-GGZ), who are diagnosed with alcohol use disorder (AUD) or cannabis use disorder (CUD). TAU follows the CBT protocol as instructed by Resultaten Scoren, which is aimed at training of and reinforcing self-control by means of self-observation, -instruction and -sense. Further, a 30% of therapy time within the GB-GGZ can be filled with therapies that are individually tailored (e.g., medicine, or therapy aimed at co-occurring symptoms).

5.3 Escape medication (if applicable)

Not applicable.

6. INVESTIGATIONAL PRODUCT

Not applicable. The current study will be testing an intervention/treatment, and not a medical product.

6.1 Name and description of investigational product(s)

6.2 Summary of findings from non-clinical studies

Not applicable

6.3 Summary of findings from clinical studies

Not applicable

6.4 Summary of known and potential risks and benefits

Not applicable

6.5 Description and justification of route of administration and dosage

Not applicable.

6.6 Dosages, dosage modifications and method of administration

Not applicable.

6.7 Preparation and labelling of Investigational Medicinal Product

Not applicable.

6.8 Drug accountability

Not applicable.

7. NON-INVESTIGATIONAL PRODUCT

Not applicable.

7.1 Name and description of non-investigational product(s)

7.2 Summary of findings from non-clinical studies

7.3 Summary of findings from clinical studies

7.4 Summary of known and potential risks and benefits

7.5 Description and justification of route of administration and dosage

7.6 Dosages, dosage modifications and method of administration

7.7 Preparation and labelling of Non Investigational Medicinal Product

7.8 Drug accountability

8. METHODS

8.1 Study parameters/endpoints

8.1.1 Main study parameter/endpoint

The primary outcome parameters are changes in substance use (i.e., quantity/frequency of use in the past 30 days), level of dependency and craving (as assessed with the MATE – measurements in addiction for triage and evaluation), and relapse-rates as measured by re-entering treatment. Further, health condition (QALY as measured by the EQ5D), and societal costs (including health care use, measured with the TiC-P) will be primary outcome parameters to assess cost-effectiveness of this intervention.

8.1.2 Secondary study parameters/endpoints (if applicable)

The secondary outcome parameters are attentional bias, and secondary complaints (anxiety, depression and stress). Further outcome parameters are subjective evaluations of the training, from the perspectives of both patient and therapist.

8.1.3 Other study parameters (if applicable)

Lastly we would like to analyse for whom this training worked and for whom it did not work (with independent variables such as co-occurring diagnoses, length of addiction period, number of sessions that were completed, motivation for treatment (N of no-show)).

8.2 Randomisation, blinding and treatment allocation

Patients will be stratified according to type of addiction (AUD or CUD), gender (M/F), age category (18-30/30-50/50+) and addiction center (VNN, Tactus, Novadic-Kentron). Within these strata's they will be randomized to one of the three intervention groups (iABM, placebo-training, no intervention). Stratification procedure is built in in the system that will send automatic invitations to the participants for the online assessments and training sessions. In this system it is guaranteed that participants will be assigned random into each study arm, ánd that number of patients in each study arm will be held approximately equal.

Patients in the no intervention condition will obviously be aware that they receive no intervention. Patients in the placebo and real condition will be uninformed about their condition. Randomization will take place by a third party, so researchers that are involved with the pre- and postmeasures will be blind for the condition of patients. Further, the therapists will be held blind for the two training versions (iABM or alternative training), so they will be blind for the condition of the patients.

The randomization code will only be broken if there will be any negative situations in patients which could be related to this study.

8.3 Study procedures

Patients will be informed about the study running by their therapist (preferably) during intake procedure or else at their first appointment of regular treatment. They will receive a short oral explanation of the study. If they are generally interested in participating in the study the therapist will hand them the patient information letter and an informed consent form (including a retour envelope; see also 11.2. for recruitment and consent procedure). Further, the therapist asks the patient if he may had their telephone number to the researchers so they can contact the patient to give him or her more information about the study.

Patients who are interested in participating will be contacted by the researchers by phone. The researcher will inform the potential participant about the study in more detail and will answer his questions. The researcher further checks whether the patient meets the inclusion and exclusion criteria. If the patient meets this criteria, and decides to participate he will be asked to sign the informed consent form and to send it back to the researchers. Because it is important to start the training as soon as the standard treatment starts, patients will be asked to decide if they would like to participate and return the signed informed consent form at their second appointment at the latest. To promote participation, patients randomized into one of the control groups will be offered the real iABM training after the end of FU2, so they will also profit from the benefits if proven effective. Standard treatment in the GB-GGZ consists of cognitive behavioural therapy (CBT) with the possibility of some additional individually adjusted treatment components (i.e., medication, or elements of anxiety/depression treatment). All participating patients will complete a pre-test, a post-test at the end of their regular treatment, and a 6-month and 12-month follow-up assessment.

Only if patients sign the consent form, they will be directed to the randomisation procedure and thereafter the online pre-test, consisting of an attentional bias task (5 minutes), two questionnaires: the EQ-5D Health Questionnaire (24), and the TiC-P (23) for costs associated with psychiatric illness (10 minutes total). The MATE (Measurements in the Addictions for Triage and Evaluation) will be assessed by the therapist or intaker before start of the regular treatment procedure. For the current project we make use of the parts 1, 3, 4 and Q1, Q2.

Patients who are assigned to the TAU condition receive their TAU as usual. Patients who are assigned to a training (iABM or placebo) + TAU condition receive their TAU as usual with the additional intervention. In the three weeks following the pretest, patients in one of the training conditions will complete a training session at home via the internet every day. Each session takes 10 minutes. The next three weeks patients

will complete a training session three times a week, and thereafter they will train once a week, for as long as the duration of TAU. The maximum amount of treatment in GB-GGZ is 750 minutes, which can be spread according to the needs of the patients. For example some patients might need more time between appointments than others. Most of the time treatment in GB-GGZ lasts for 3 to 4 months. Before and after each training session participants will score their craving on a VAS (visual analogue scale). This VAS is built in in the training programme. Further, compliance will be automatically monitored, by following if, when and in which time-frame participants completed each training session. After the first week of training (and again after 6 weeks) participants will be asked to fill in some extra questions to evaluate compliance and the perceived credibility of the training.

At the end of the last intervention, which coincides with the end of TAU, patients complete the post-test, including the same measures as the pre-test. The MATE (25) will be assessed as a standard routine outcome measurement (ROM) by the therapist post-treatment (which coincides with the post-intervention moment). We will use the parts 1, 4 and Q1, Q2 for the current project. Further, therapists will be asked to fill-in a short evaluation form regarding their time investment in this study and possible direct positive or negative side effects they experienced from the training.

Six and 12 month after finishing the intervention and TAU, patients will receive a follow-up online assessment consisting of the attentional bias task, the parts 1, 4, Q1 and Q2 of the MATE, and the EQ5D and Tic-P.

The pre, post and FU online assessments follow the same procedure of approximately 20 minutes and use the following measures:

- Attentional bias: Odd-one out task (26, 27, 28, 29). Initially we wanted to use another assessment task, but this task was found to be too difficult for many participants in our pilot study. Therefore, we decided to use this easy and simple AB task, which has yielded good validity in previous research.
- Perceived health condition: EQ5D (EuroQol)(24)
- Use of health care, sickness and work: Tic-P(23)

The 6- and 12-month FU measures also include the parts 1,4, Q1 and Q2 of the MATE (25) (the attached file is a pdf of parts 1, 4, Q1 and Q2 of the original MATE form. For the pre and post measure these data will be taken from the electronic patient system, and added to the other research data under pseudonym in the research dossier. Patients will be asked to agree on the informed consent form that the MATE data (of parts 1,4, Q1 and Q2) can be used for the current research. For the 6 and 12 month FU the questionnaire will be available online (under participant number, thus no link to patient numbers will be made).

8.4 Withdrawal of individual subjects

Subjects can leave the study at any time for any reason if they wish to do so without any consequences. The investigator can decide to withdraw a subject from the study for urgent medical reasons.

8.4.1 Specific criteria for withdrawal (if applicable)

8.5 Replacement of individual subjects after withdrawal

Subjects will not be replaced after withdrawal – we accounted for drop-out in the sample size calculation.

8.6 Follow-up of subjects withdrawn from treatment

For completeness of results, subjects who withdraw from the treatment will still be approached to participate in post- and FU assessments.

8.7 Premature termination of the study

There are no criteria on the basis of which we expect the study to be terminated. If inclusion of patients is slower than expected, we will focus on how we will be able to increase the number of participants. It might be that motivating the therapists is helpful. An other possibility is that we will invite other addiction treatment centres to participate in the study.

9. SAFETY REPORTING

9.1 Temporary halt for reasons of subject safety

In accordance to section 10, subsection 4, of the WMO, the sponsor will suspend the study if there is sufficient ground that continuation of the study will jeopardise subject health or safety. The sponsor will notify the accredited METC without undue delay of a temporary halt including the reason for such an action. The study will be suspended pending a further positive decision by the accredited METC. The investigator will take care that all subjects are kept informed.

9.2 AEs, SAEs and SUSARs

9.2.1 Adverse events (AEs)

Adverse events are defined as any undesirable experience occurring to a subject during the study, whether or not considered related to the iABM. All adverse events reported spontaneously by the subject or observed by the investigator or his staff will be recorded.

9.2.2 Serious adverse events (SAEs)

A serious adverse event is any untoward medical occurrence or effect that

- results in death;
- is life threatening (at the time of the event);
- requires hospitalisation or prolongation of existing inpatients' hospitalisation;
- results in persistent or significant disability or incapacity;
- is a congenital anomaly or birth defect; or
- any other important medical event that did not result in any of the outcomes listed above due to medical or surgical intervention but could have been based upon appropriate judgement by the investigator.

An elective hospital admission will not be considered as a serious adverse event.

The investigator will report all SAEs to the sponsor without undue delay after obtaining knowledge of the events.

The sponsor will report the SAEs through the web portal *ToetsingOnline* to the accredited METC that approved the protocol, within 7 days of first knowledge for SAEs that result in death or are life threatening followed by a period of maximum of 8 days to complete the initial preliminary report. All other SAEs will be reported within a

period of maximum 15 days after the sponsor has first knowledge of the serious adverse events.

9.2.3 Suspected unexpected serious adverse reactions (SUSARs)

Not applicable.

9.3 Annual safety report

Not applicable.

9.4 Follow-up of adverse events

All AEs will be followed until they have abated, or until a stable situation has been reached. Depending on the event, follow up may require additional tests or medical procedures as indicated, and/or referral to the general physician or a medical specialist. SAEs need to be reported till end of study within the Netherlands, as defined in the protocol

9.5 [Data Safety Monitoring Board (DSMB) / Safety Committee]

Not applicable.

10. STATISTICAL ANALYSIS

All data will be presented quantitative.

If drop-out rates are larger than 5% of the total data we will multiple impute the dataset to account for missing variables. We will apply multiple imputation in order to estimate the FU missing-data as a state-of-the-art method for dealing with missing data (e.g., 30). We will impute the missing data in SPSS using M = 40 imputations, and to avoid bias due to missingness we will use baseline variables that might be predictive for missingness at FU (age, gender, severity of substance dependency, AB) as indicators in the model (31). We will use this imputed data-set for the analyses and will report the pooled results. Further, ITT analysis will be conducted using the complete sample. To correct for multiple comparisons we will use the FDR (false discovery rate; 32).

10.1 Primary study parameter(s)

To examine the effects of the iABM on substance use and craving, we will conduct a 4 (within subjects: pre, post, FU1, FU2) x 2 (between subjects: iABM+TAU versus placebo+TAU or TAU only condition) repeated measure ANOVAs, with substance use and craving as dependent variables. Thus, participants' pre-test scores will be taken into account in this analysis, which will reveal treatment-induced improvement. A post-hoc analysis will be performed to compare the iABM group with the each control group (thus iABM+TAU compared to placebo+TAU, and iABM+TAU compared to TAU only). Next to this, ITT analysis will be performed in which also the drop-outs will be taken into account.

Further, the effects of the training on relapse rates, will be analysed by means of a Cox regression, to model the time it takes for a relapse to occur.

Further, the effect of the training on health condition will be tested by a 4 (within subjects: pre, post, FU1, FU2) x 2 (between subjects: iABM+TAU versus placebo+TAU or TAU only condition) repeated measure ANOVAs, with health condition as dependent variable. Thus, participants' pre-test scores will be taken into account in this analysis, which will reveal treatment-induced improvement.

10.2 Secondary study parameter(s)

Secondary outcome measure of AB will be tested using a 4 (within subjects: pre, post, FU1, FU2) x 2 (between subjects: : iABM+TAU versus placebo+TAU or TAU only condition) repeated measure ANOVAs, with attentional bias as dependent variable. We

further will test whether change in symptoms is mediated by a change in AB and/or craving, using a mediation regression model via path modelling.

10.3 Other study parameters

Primary outcome measures that will be included in the planned cost-effectiveness analyses (CEA) are substance use and relapse rates. Furthermore, a cost-utility analysis will be conducted with the QALY (Quality Adjusted Life Years) as primary outcome measure (derived from the EQ-5D). Costs and health outcomes will not be discounted due to the current time horizon of 12 months. Uni- and multivariate sensitivity analyses will address important cost aspects, including for instance components of the intervention costs and contacts with healthcare. The uncertainty surrounding the cost-effectiveness and cost-utility ratios will be assessed by bootstrap analyses. In addition, cost-effectiveness acceptability curves will be used to inform decision-makers on the probability that the examined intervention is cost-effective.

A budget impact analysis (BIA) based on trial results in combination with epidemiological analysis will provide insight into the number of potential patients in the Netherlands that would start to use iABM. Costs and savings will be estimated based on the cost estimates of the trial and extrapolated to a longer time horizon using modeling. Due to the large uncertainty involved in the latter, two extreme scenarios will be compared. One with no difference in costs after the study follow-up period, and one that extrapolates observed cost-differences over a longer time horizon of 4 years, assuming costs remain stable at the level measured at 12 months follow-up.

10.4 Interim analysis (if applicable)

Not applicable.

11. ETHICAL CONSIDERATIONS

11.1 Regulation statement

The *study will be conducted according to the principles of the Declaration of Helsinki* 64th WMA General Assembly, Fortaleza, Brazil, October 2013) *and in accordance with the Medical Research Involving Human Subjects Act (WMO).*

11.2 Recruitment and consent

Patients will be informed about the study by their therapists, when they are referred to the GB-GGZ at one of the three addiction centres. The therapists will give a short oral explanation about the research project. If the patient is generally interested in participating in the study, the therapist will hand them the patient information letter and an informed consent (including a retour envelope). Thereby we want to make sure that the potential participant has enough time to read the information at home. Furthermore the therapist will ask the patient whether he agrees with passing his name and phone number to the researchers. If the patient prefers to contact the researchers himself, the therapist will hand him the phone number of the researchers. If the patient agrees, the therapist will forward the personal data via a secure electronical system to the researchers. Then the researcher will contact the potential participant by phone in order to inform him about the content, aim and duration of the study; the possible health risk's; the possible risk's due to early drop out of the study; and other possible objections due to participating in the study. If thereafter the patient has no more questions the researcher will ask him whether he already made a decision about participating in the study. If the potential participant needs more time to think about participation, the researcher will make a new appointment by telephone. If the potential participant already decided to participate in the study, he is asked to sign the informed consent (which he got from his therapist together with the patient information letter) and to send it as soon as possible to the researchers, using the retour envelope. If the patient lost the informed consent the researcher will send a new one to the potential participant via post. After the researchers received the signed informed consent the participant will be assigned to the system of the third party for randomization and will receive an invitation for the pre-measurement via email.
In total the participants will be given one to maximum two weeks (in between intake and first or at the latest the second appointment of regular treatment) to consider whether they will participate in the study. That's because pre-measurements could otherwise be influenced by early effects of the regular treatment sessions.

11.3 Objection by minors or incapacitated subjects (if applicable)

Not applicable.

11.4 Benefits and risks assessment, group relatedness

The potential risks and burdens of this study are rather low related to the expected benefits. That is, 75% of the patients are receiving a training for which we expect at least some effect on substance use (problems). We expect that patients in the real iABM condition (and it is to be expected that this to a lesser extent also yields for patients in the alternative training condition) experience direct effect on their craving for alcohol/cannabis, and longer-term effect on abstinence (lower relapse risk). We expect that these patients (especially in the iABM condition) with a little effort (because training can be done at home, and is relatively easy and short) receive this positive effect on their problems and on their quality of life. The remaining 25% still receives TAU, thus there are no patients refrained from therapy, and most of the patients are expected to benefit from the additional training. It might however be that craving will increase by being confronted with pictures of ‘their’ substance at these moments that craving already is high. However, to our knowledge, there are no studies that reported such a negative side effect from this kind of experimental tasks or trainings (only decrease of craving, e.g., 18). We therefore do not expect a negative side-effect like this to occur. Further, with alcohol and drugs being everywhere around in ‘normal life’ we expect that the confrontation with only pictures would be less craving-evoking than the real life situations. However, during the project the researchers and therapists will be in close contact, to be able to anticipate on any negative (unexpected) situation. Further, participants are allowed to continue care as usual and participation is voluntary. Participants will be informed about the study and will have full freedom to withdraw from the study at any point and for any reason, if they wish to.

Summarizing, we expect a direct and longer-term benefit for both training groups, related to a small investment (short sessions, at home environment), and no potential risk factors. Further, we expect no extra benefit on top of TAU for the control group, related to a minimum investment of four times a 20-30 minute assessment, and no potential risk factors.

11.5 Compensation for injury

The intervention that will be tested in this project is of a low-risk nature because patients do not need to extra visit the organisation, they need no medical procedures or need to take medicines for this project. Previous ABM research in the field of addiction (and anxiety, depression) did not report any serious adverse effects of ABM training (e.g., an increasing effect on craving). Therefore, we expect no increased risk of the current experimental

intervention. Because of the low risk of the current intervention, dispensation of insurance is being requested.

11.6 Incentives (if applicable)

Not applicable.

12. ADMINISTRATIVE ASPECTS, MONITORING AND PUBLICATION

12.1 Handling and storage of data and documents

All data received from participants will be processed in a strictly confidential manner. Data will be stored and coded in a numerical way under number of inclusion. The project leader, Dr. M.E. van Hemel-Ruiter and the principal investigator, J. Heitmann (MSc.) know this code. Access to documents and possible other information that can be traced back to participants is only available to the project leader and the principal investigator.
This method of data coding and code access will ensure the privacy of participants. Data used for publication are also completely anonymous. All handling of personal data will comply with the Dutch Personal Data Protection Act (Wet Bescherming Persoonsgegevens). To be able to couple the data to the MATE data from the Electronic Patient System (EPD) the data will get pseudonyms first. After this, data will get an anonym identification number. Data will be stored for 15 years. Currently a data-management is prepared and will be finished before start of the inclusion of patients.

12.2 Monitoring and Quality Assurance

The principal investigator will monitor the conduct of the study following the guidelines of ZonMw. This will include the description of the project, the current METc request, the research protocol, logbook, code books, and the individuals involved in the project. This will all be described in the data-management plan. The preliminary plan you will find attached. We would like to stress that agreements are made with ZonMw, and data-management plan will be finished in Februari 2016.

12.3 Amendments

A ‘substantial amendment’ is defined as an amendment to the terms of the METC application, or to the protocol or any other supporting documentation, that is likely to affect to a significant degree:

- the safety or physical or mental integrity of the subjects of the trial;
- the scientific value of the trial;
- the conduct or management of the trial; or
- the quality or safety of any intervention used in the trial.

All substantial amendments will be notified to the METC and to the competent authority.

Non-substantial amendments will not be notified to the accredited METC and the competent authority, but will be recorded and filed by the sponsor.

12.4 Annual progress report

The sponsor/investigator will submit a summary of the progress of the trial to the accredited METC once a year. Information will be provided on the date of inclusion of the first subject, numbers of subjects included and numbers of subjects that have completed the trial, serious adverse events/ serious adverse reactions, other problems, and amendments.

12.5 Temporary halt and (prematurely) end of study report

The investigator/sponsor will notify the accredited METC of the end of the study within a period of 8 weeks. The end of the study is defined as the last patient's last 12-month follow-up assessment.

The sponsor will notify the METC immediately of a temporary halt of the study, including the reason of such an action.

In case the study is ended prematurely, the sponsor will notify the accredited METC within 15 days, including the reasons for the premature termination.

Within one year after the end of the study, the investigator/sponsor will submit a final study report with the results of the study, including any publications/abstracts of the study, to the accredited METC.

12.6 Public disclosure and publication policy

The insights resulting from this study will be disseminated to the following main target groups:

- Psychologists/ therapists: they need to be trained in assisting the patients in optimally using the iABM therapy. They need to be capable in motivating the patients in completing the iABM-sessions
- Patients, who need to know that iABM is a relatively simple and patient friendly therapy that will help them to successfully master their alcohol or cannabis use disorder
- Researchers in the field of treating alcohol and cannabis use disorders
- General practitioners: they need to know of the iABM-therapy and why it might be relevant for their patients so they can activate/motivate them to use it. So they need to be aware of the (cost-)effectiveness and the patient friendliness of iABM
- Policy makers and health insurance companies

Publications in core medical and psychological journals (international and national) will target health care professionals in the area of addiction care. Besides, the results of this study

will be presented at both national and international meetings in the addiction field and at meetings of other relevant stakeholders, such as patient associations, the financing partners and the Dutch Health Inspectorate (IGZ).

13. STRUCTURED RISK ANALYSIS

Not applicable

13.1 Potential issues of concern

a. Level of knowledge about mechanism of action

b. Previous exposure of human beings with the test product(s) and/or products with a similar biological mechanism

c. Can the primary or secondary mechanism be induced in animals and/or in *ex-vivo* human cell material?

d. Selectivity of the mechanism to target tissue in animals and/or human beings

e. Analysis of potential effect

f. Pharmacokinetic considerations

g. Study population

h. Interaction with other products

i. Predictability of effect

j. Can effects be managed?

13.2 Synthesis

14. REFERENCES

1 Suijkerbuijk AWM, van Gils PF, de Wit GA (2014). De kosteneffectiviteit van interventies gericht op verslaving aan alcohol en middelen : Een review van de literatuur. RIVM rapport.

2 Polman & Visser, 2014. Verslavingszorg in beeld. Alcohol en drugs. Zorginstituut Nederland.

3 Deutsch, R., & Strack, F. (2006). Reflective and impulsive determinants of addictive behavior. In R. W. Wiers, A. W. Stacy, R. W. Wiers & A. W. Stacy (Eds.), *Handbook of implicit cognition and addiction* (pp. 45-57). Thousand Oaks, CA US: Sage Publications, Inc.

4 Franken, I. H. A. (2003). Drug craving and addiction: Integrating psychological and neuropsychopharmacological approaches. *Progress in Neuro-Psychopharmacology & Biological Psychiatry, 27*(4), 563-579.

5 Lubman, D. I., Peters, L. A., Mogg, K., Bradley, B. P., & Deakin, J. F. W. (2000). Attentional bias for drug cues in opiate dependence. *Psychological Medicine: A Journal of Research in Psychiatry and the Allied Sciences, 30*(1), 169-175.

6 Field, M., & Cox, W.M. (2008). Attentional bias in addictive behaviors: A review of its development, causes and consequences. *Drug and Alcohol Dependence, 97 (1-2):* 1-20.

7 Fadardi, J.S., & Cox, W.M. (2006). Alcohol attentional bias: drinking salience or cognitive impairment? Psychopharmacology 185 (2), 169-178

8 Noël, X., Colmant, M., Van, D. L., Bechara, A., Bullens, Q., Hanak, C., et al. (2006). Time course of attention for alcohol cues in abstinent alcoholic patients: The role of initial orienting. *Alcoholism: Clinical and Experimental Research, 30*(11), 1871-1877.

9 Carpenter, K. M., Schreiber, E., Church, S., & McDowell, D. (2006). Drug stroop performance: Relationships with primary substance of use and treatment outcome in a drug-dependent outpatient sample. *Addictive Behaviors, 31*(1), 174-181.

10 Cox, W. M., Hogan, L. M., Kristian, M. R., & Race, J. H. (2002). Alcohol attentional bias as a predictor of alcohol abusers' treatment outcome. *Drug and Alcohol Dependence, 68*(3), 237-243.

11 Marissen, M. A. E., Franken, I. H. A., Waters, A. J., Blanken, P., van, d. B., & Hendriks, V. M. (2006). Attentional bias predicts heroin relapse following treatment. *Addiction, 101*(9), 1306-1312.

12 Cox, W. M., Pothos, E. M., & Hosier, S. G. (2007). Cognitive-motivational predictors of excessive drinkers' success in changing. *Psychopharmacology, 192*(4), 499-510.

13 MacLeod, C., Rutherford, E., Campbell, L., Ebsworthy, G., & Holker, L. (2002). Selective attention and emotional vulnerability: Assessing the causal basis of their association through the experimental manipulation of attentional bias. *Journal of Abnormal Psychology, 111*(1), 107-123

14 Fadardi, J.S., & Cox, W.M. (2009). Reversing the sequence: reducing alcohol consumption by overcoming alcohol attentional bias. *Drug and Alcohol Dependence, 101 (3),* 137-145.

15 Schoenmakers, T.M., de Bruin, M., Lux, I.F., Goertz, A.G., Van Kerkhof, D.H., & Wiers, R.W. (2010). Clinical effectiveness of attentional bias modification training in abstinent alcoholic patients. *Drug and Alcohol Dependence, 109,* 30-36.

16 McGeary, J. E., Meadows, S. P., Amir, N., & Gibb, B. E. (2014). Computer-delivered, home-based, attentional retraining reduces drinking behavior in heavy drinkers. Psychology of Addictive Behaviors : Journal of the Society of Psychologists in Addictive Behaviors, 28(2), 559-562.

17 Schoenmakers, T. M., Bruin, M., Lux, I. F., Goertz, A. G., Kerkhof, D. H., & Wiers, R. W. (2010). Clinical effectiveness of attentional bias modification training in abstinent alcoholic patients. Drug and Alcohol Dependence, 109(1-3), 30-36.

18 Kerst, W. F., & Waters, A. J. (2014). Attentional retraining administered in the field reduces smokers' attentional bias and craving. Health Psychology : Official Journal of the Division of Health Psychology, American Psychological Association, 33(10), 1232-1240.

19 Fadardi JS, Cox WM. Reversing the sequence: reducing alcohol consumption by overcoming alcohol attentional bias. Drug and Alcohol Dependence. 2009;101:137–145.
20 Christiansen, Schoenmaker & Field, 2015. Less than meets the eye: Reappraising the clinical relevance of attentional bias in addiction. Addictive behaviors.

21 Grafton, B., Notebaert, L. & MacLeod, C. (in preparation). Emotion in motion: A novel approach to the assessment and modification of attentional bias.

22 Kuckertz, J. M., Gildebrant, E., Liliequist, B., Karlstrom, P., Vappling, C., Bodlund, O., et al. (2014). Moderation and mediation of the effect of attention training in social anxiety disorder. *Behaviour Research and Therapy, 53*, 30-40.

23 Hakkaart-van Roijen L(2002). Trimbos/iMTA questionnaire for Costs associated with Psychiatric Illness (TiC-P). Rotterdam, the Netherlands: Erasmus University (in Dutch).

24 Dolan P (1997). Modeling valuations for EuroQol health states. *Medical Care,* 35(11):1095-108.

25 Schippers, G.M. & Broekman, T.G. (2007). MATE, Measurements in the Addictions for Triage and Evaluation. Development of an instrument assessing patient characteristics in substance abuse treatment. Final report ZonMw/Resultaten Scoren-project nr 31000068. Amsterdam, AIAR, AMC-Amsterdam & Bureau Bêta, Nijmegen.

26 Oliver & Drobes, 2012. Visual search and attentional bias for smoking cues: The role of familiarity. Experimental and Clinical Psychopharmacology, Vol 20(6), 489-496.

27 Smeets, Roefs, Furth & Jansen, 2008. Experimentally induced chocolate craving leads to an attentional bias in increased distraction but not in speeded detection. Appetite 53(3):370-5.

28 Dodd, H. F., Vogt, J., Turkileri, N., & Notebaert, L. (2016). Task relevance of emotional information affects anxiety-linked attention bias in visual search. Biological Psychology,

29 Rinck, M., Reinecke, A., Ellwart, T., Heuer, K., & Becker, E. S. (2005). Speeded detection and increased distraction in fear of spiders: Evidence from eye movements. Journal of Abnormal Psychology, 114(2), 235-248.

30 Jeličić, H., Phelps E., Lerner, R.M., 2010. Why missing data matter in the longitudinal study of adolescent development: Using the 4-H study to understand the uses of different missing data methods. J Youth Adolesc. 39, 816–835.

31 Sterne, J.A.C., White, I.R., Carlin, J.B., Spratt, M., Royston, P., Kenward, M.G., Wood, A.M., Carpenter, J.R., 2009. Multiple imputation for missing data in epidemiological and clinical research: potential and pitfalls. BMJ. 338.

32 Benjamini en Hochberg, Controlling the False Discovery Rate: a practical and powerful approach to multiple testing (Journal Royal Stat. Soc. B, 1995)
